# Optimization of electrical stimulation for the treatment of lower limb dysfunction after stroke: A systematic review and Bayesian network meta-analysis of randomized controlled trials

**DOI:** 10.1371/journal.pone.0285523

**Published:** 2023-05-11

**Authors:** Yu Fang, Jiang Li, Shanyu Liu, Yan Wang, Jiaming Li, Dongdong Yang, Qiaoling Wang

**Affiliations:** 1 Department of Neurology, Hospital of Chengdu University of Traditional Chinese Medicine, Chengdu, China; 2 General Practice Department, Hospital of Chengdu University of Traditional Chinese Medicine, Chengdu, China; 3 Department of Neurology, The Fifth People’s Hospital of Chengdu/ The Fifth Affiliated Hospital of Chengdu University of traditional Chinese Medicine, Chengdu, China; 4 Department of Ministry of Science, Hospital of Chengdu University of Traditional Chinese Medicine, Chengdu, China; Nnamdi Azikiwe University, NIGERIA

## Abstract

**Objective:**

To compare the treatment effect of five electrical stimulation methods commonly used in the treatment of stroke patients with lower limb dysfunction.

**Methods:**

We implemented a systematic search of 3915 studies published up to January 2023 from eight databases and two clinical trial registries. First, two independent reviewers critically evaluated trial eligibility according to the inclusion and exclusion criteria. Next, they selected and extracted data. Then, they assessed the risk of bias. Pairwise meta-analysis and Bayesian network meta-analysis were conducted to estimate the effectiveness and ranking of the five electrical stimulation methods.

**Results:**

A total of 33 trials with a final total of 2246 subjects were included in the analysis. By combining the comprehensive Rehabilitation Treatment (RT), the treatment effects of using five electrical stimulation methods were surperior to those of using RT only. In the meantime, RT+transcranial Direct Current Stimulation(tDCS) and RT+Functional Electrical Stimulation(FES) could be the optimal electric stimulation schemes for restoring lower limb motor function(SMD 8.35, 95%CI [3.05, 13.34]/ SMD 5.64, 95%CI [3.68, 7.56]), improving balance (SMD 9.80, 95%CI [0.67, 20.93]/ SMD 6.54, 95%CI [3.85, 10.95]) and activities of daily living(SMD 18.95, 95%CI [0.401, 36.9]/ SMD 15.47, 95%CI [7.89, 22.75]), and the treatment effects would be even better using RT+FES+tDCS combination.

**Conclusion:**

tDCS and FES superior to other electrical stimulation methods based on RT in the treatment of lower limb dysfunction after stroke.

## Introduction

Stroke is a disorder of cerebral blood circulation that can lead to neurological deficits [1, 2]. Previous studies indicated that the proportion of stroke in the global burden of the disease will increase year by year [3]. Stroke holds the characteristics of high incidence rate, high disability rate, high mortality, high recurrence rate, high economic burden and so on [4], which has led to a growing number of elderly people being disabled [5]. Stroke-induced lower limb dysfunction mainly affects the walking ability of patients, 63% of patients lose walking ability in the early post-stroke period and 22% cannot walk independently even after clinical and comprehensive rehabilitative interventions [6]. Lower limb dysfunction not only seriously impacts patients’ daily life but also brings serious mental and economic stress to patients and their families [7]. Therefore, establishing scientific and effective rehabilitation treatment schemes are crucial for such patients.

With the increasing understanding of lower limb dysfunction after stroke, various rehabilitation therapies have been applied to restore motor function, including traditional rehabilitation therapies such as occupational therapy, exercise therapy, and mirror therapy [8]. Emerging technologies like virtual reality [9], brain-computer interface technology [10], and intelligent robot training [11] have also been adopted in rehabilitation therapy. Nevertheless, few studies have demonstrated the clinical effectiveness of the above new methods. In addition, due to the lengthy rehabilitation process, economic benefits have become a high priority for clinicians and patients when choosing treatment methods [12].

In recent years, electrical stimulation has been widely used to improve limb function after stroke through clinical practice [13]. Considerable clinical trials have been implemented on electrical stimulation for the treatment of lower limb dysfunction after stroke [13]. The frequently-used electrical stimulation methods in the treatment of lower limb dysfunction after stroke include transcranial Direct Current Stimulation (tDCS), Neuromuscular Electrical Stimulation (NMES), Functional Electrical Stimulation (FES), Transcutaneous Electrical Nerve Stimulation (TENS), and Transcutaneous Electrical Acupoint Stimulation (TEAS) [14]. TENS is an electrical stimulation method adopting transdermal output pulse current to efficiently relieve the pain and stimulate the sensory impulses, thus improving muscle strength and motor function, meanwhile reducing spasticity [15] TEAS is a method of electrical stimulation of acupuncture points by TENS under the guidance of the meridian and acupoint theory of Chinese medicine [16]. NMES typically applies higher frequencies (20–50 Hz) current to promote muscle strength and relieve spasm symptoms [17]. FES is the most commonly used electrical stimulation methods for the treatment of lower limb dysfunction, and its basic principle is the simultaneous or intermittent use of electrical stimulation combined with functional tasks [18], from which a series of FES-based rehabilitation therapies have been derived. The tDCS is the only electrical stimulation method whose stimulation site is in the head, and its mechanism may be that it has short-term and long-term effects on cortical excitability and neuroplasticity [19]. In our previous study [20], the effectiveness of the above-mentioned five electrical stimulation methods in the treatment of upper limb dysfunction after stroke has been evaluated. In the follow-up work, we found that considerable efforts have been devoted to systematic review and meta-analysis on the effects of the abovementioned methods in the treatment of lower limb dysfunction after stroke. Nascimento et al [21] compared the effects of ankle-foot orthosis and FES on patients’ walking speed. Bai et al [22] investigated the ability of tDCS to restore motor function in patients’ lower limbs. Hong et al [23] investigated the effects of NMES on lower limb motor function after stroke. So far, nevertheless, the previous studies have been dedicated to exploring the effectiveness of a single electrical stimulation method, and few effort has been made to the comprehensive comparative analysis of various electrical stimulation methods). To address this issue, we adopted network meta-analysis [24] to select subjects with ischemic or hemorrhagic stroke and lower limb dysfunction based on high-quality randomized controlled trials. Combined with routine comprehensive rehabilitation therapy (RT), 5 outcome measures (FMA-LE, BBS, MBI, CSS and 10mMWS) were applied to evaluate the treatmnet effect of five different electrical stimulation schemes (RT+FES, RT+NMES, RT+TENS, RT+TEAS, RT+tDCS) on lower limb dysfunction after stroke, and the guiding significance of the results to clinical practice was discussed.

## Methods

This study followed the PRISMA-NMA guidelines [25] (shown in S1 Table). The study has been registered in the Open Science Framework (registration DOI:10.17605/OSF.IO/F3G5Q).

### Search strategy

We conducted an exhaustive online search for eligible studies by setting the retrieval time from the establishing date of each database to January 7th, 2023. The literature language was limited within English and Chinese. We searched in eight electronic databases, including China National Knowledge Infrastructure (CNKI), VIP Database for Chinese Technical Periodicals (VIP), WAN FANG Database (WF), Chinese biomedical literature service system (SinoMed), PubMed, Web of Science (WOS), Embase, and Cochrane Library. The clinical trial registries consisted of the International Standard Randomized Controlled Trial Number Register (ISRCTN) and the Chinese Clinical Trial Registry (ChiCTR). The MeSH terms used in this study included: Hemiplegia, Paralysis, Clinical trials as topic, Stroke, Electric Stimulation et al. We set four categories of free words, including (1) Lower limbs, leg, foot; (2) Motor function, Hemiplegia, Dysfunction; (3) Randomized controlled trial, controlled clinical trial, clinical trials; and (4) Stroke, Cerebrovascular Accident, Brain Vascular Accident, etc. For an example, the PubMed search strategies is shown in Table 1, and the search iterms were appropriately adjusted to meet the requirements of each database in order to ensure the basic logical integrity of the search.

**Table 1:** Data retrieval strategy for PubMed database.

Steps	Search
#1	(Electric Stimulation[MeSH Terms]) OR (Electric Stimulation Therapy[Title/Abstract]) OR (Electrotherapy[Title/Abstract]) OR (transcutaneous electrical nerve stimulation[Title/Abstract]) OR (Transcutaneous Electric Stimulation[Title/Abstract]) OR (Percutaneous Electric Nerve Stimulation[Title/Abstract]) OR (TENS[Title/Abstract]) OR (Transcutaneous Electrical Stimulation[Title/Abstract]) OR (Transdermal Electrostimulation[Title/Abstract]) OR (transcutaneous electrical acupoint stimulation[Title/Abstract]) OR (TEAS[Title/Abstract]) OR (neuromuscular electrical stimulation[Title/Abstract]) OR (NMES[Title/Abstract]) OR (functional electrical stimulation[Title/Abstract]) OR (FES[Title/Abstract]) OR (Transcranial Direct Current Stimulation[Title/Abstract]) OR (tDCS[Title/Abstract])
#2	(Stroke[MeSH Terms]) OR (Cerebrovascular Accident[Title/Abstract]) OR (CVA[Title/Abstract]) OR (Brain Vascular Accident[Title/Abstract]) OR (Apoplexy[Title/Abstract])
#3	(Randomized controlled trial[Publication Type]) OR (Controlled clinical trial[Publication Type]) OR (Randomized[Title/Abstract]) OR (Clinical trials as topic[MeSH Terms]) OR (Randomly[Title/Abstract]) OR (Trial[Title]) OR (Clinical[Title])
#4	(Hemiplegia[MeSH Terms]) OR (Paralysis[MeSH Terms]) OR (Motor function[Title/Abstract]) OR (Dysfunction[Title/Abstract]) OR (Lower limbs[Title/Abstract]) OR (Lower extremities[Title/Abstract]) OR (Leg[Title/Abstract]) OR (Digit[Title/Abstract]) OR (Toe[Title/Abstract]) OR (Knee[Title/Abstract]) OR (Ankle[Title/Abstract]) OR (Foot[Title/Abstract]) OR (Thigh[Title/Abstract]) OR (Lower limb[Title/Abstract]) OR (Lower extremity[Title/Abstract])
#5	#1 AND #2 AND #3 AND #4

### Inclusion and exclusion criteria

#### Study type

We screened studies that strictly met the design requirements for randomized controlled trials, including peer-reviewed journals in Chinese or English, and master and doctoral theses. Conference articles, newspaper articles, or book abstracts were excluded. We tracked studies retrieved in the Clinical Trials Registry Platform and excluded studies that were still in progress or had incomplete trial data. In addition, the treatment effects of interventions were easily exaggerated and may result in false positives due to the lack of validity of small trials that included a small number of patients [26]. In contrast, some small sample pretests usually adjusted the trial protocol or even did not conduct a formal trial in case of unsatisfactory trial results. Hence, aiming at guaranteeing the overall quality of the included studies and decreasing the bias of the present study, we excluded studies with total sample size less than 30.

#### Type of subjects

The stroke diagnostic criteria included in RCTs need to record, and the participant should meet the diagnosis of ischemic or hemorrhagic stroke with definite lower limb dysfunction. Other systemic diseases or various causes of lower limb dysfunction were excluded. In addition, this study did not restrict the age, sex, race, or course of disease of the patients.

#### Type of interventions

Since the kinds of electrical stimulation and rehabilitation treatments are various, we established strict inclusion criteria for interventions to obtain accurate literature screening results: (1) Experimental group: electrical stimulation methods including TENS, TEAS, NMES, tDCS, and FES were used combined with RT. The five electrical stimulation methods can be used separately or in combination with no restrictions on the dose, frequency, duration, or site of stimulation. (2) Control group: five electrical stimulation methods (same as the experimental group), RT, and Sham Stimulation (SS) were adopted in control group. The above interventions can be used separately or in combination. Because of the uncontrolled progression of stroke patients during rehabilitation and the complexity of their condition, the rehabilitation therapies used in each RCT were not identical. Based on the authoritative guidelines [8, 27] and clinical practice experience, the scope of RT was determined as: (1) conventional comprehensive rehabilitation techniques: exercise therapy, occupational therapy, rehabilitation training, rehabilitation education, and functional exercise; (2) conventional stroke care; (3) conventional stroke pharmacotherapy. It should be noted that we excluded RCTs using a single rehabilitation technique (e.g., mirror therapy, walking training, etc.) as an intervention, because the clinical efficacy of using different electrical stimulation methods based on comprehensive RT was the research priority of this study.

#### Type of outcome measures

The primary outcome measure of this study was the Fugl-Meyer Assessment for Lower Extremity (FMA-LE), and secondary outcome measures included the Modified Barthel Index (MBI), Berg Balance Scale (BBS), 10m Maximal Walking Speed (10mMWS), and Composite Spasticity Scale (CSS). The FMA-LE is a widely recommended and used reliable scale for assessing lower limb motor deficits after stroke and can be utilized as a reliable tool for comprehensive evaluation of lower limb motor function [28]. BBS is a reliable and valid clinical scale that is often employed to assess patients’ balance. MBI is used to evaluate the improvement of patients’ daily living ability and the ability to function independently. The scales frequently-used to evaluate patients’ increased muscular tension include MAS and CSS. For lower limbs, CSS can effectively reflect the state of ankle plantar flexor tension compared with MAS [29]. Considering that walking ability is an important factor in measuring lower limb motor function [30], 10mMWS was included as an assessment index.

### Study selection

First, two trained reviewers (YF and JL) independently screened the titles and abstracts according to inclusion criteria. Then, two reviewers assessed the full texts of potentially eligible studies. We marked the studies that were not accessible or the data was incomplete and we contacted the researcher by email. Finally, we excluded these studies after being unable to contact the researchers for three consecutive times or confirming that the studies were not accessible. Additionally, if there were divergences between the two reviewers, the third professional reviewer (QW) would intervene in for further evaluation.

### Data process and analysis

Data extraction was conducted by two independent reviewers (YW and SL). The third reviewer (QW) would intervene in if there were divergences in the data extraction process. The extracted data included study title, author name, publication date, country, disease course, sample size, gender, age, intervention, treatment period, and outcome indicators (mean and standard deviation), etc. If the interventions of different groups in the multi-arms study are the same (different courses of treatment, frequencies, etc.), we will combine the data of the two groups. Meanwhile, the two reviewers also sorted out the parameter setting information of different electrical stimulation methods in each study.

Review Manager (Revman V5.3) and Aggregate Data Drug Information System (ADDIS V1.16.8) were used to conduct the meta-analysis. Pairwise Meta-Analysis was employed to compare two interventions comprehensively. In this study, Standard Mean Differences (SMD) was used for continuous outcomes, 95% represented confidence intervals (CIs). If there was no significant heterogeneity (I^2^<50%), the fixed effect model would be used. Whereas if there existed obvious heterogeneity between studies (I^2^≥50%), the random effect model would be adopted. NMA was performed in ADDIS using the Markov chain Monte Carlo method. NMA network plots for the five outcome measures were generated in Stata software (V16.0 MP). In a network, the larger the node represents the more studies using this intervention method. The thicker the lines between nodes represents the more comparative studies involving the two intervention methods. For any possible scenario, NMA was performed only when different interventions were connected in a network (There were direct or indirect links between different interventions). Furthermore, the node-splitting method was conducted to divide the evidence for each comparison of different interventions into direct and indirect evidence to evaluate local inconsistency. The internal consistency of the evidence network determined the validity of the NMA results, and the sources of direct and various indirect evidence should be consistent [24]. The segmented node method was used in this study to test the inconsistency in the NMA. Finally, the different electrical stimulation methods were ranked using the surface under the cumulative ranking curve (SUCRA).

### Risk of bias assessment

The Cochrane risk of bias tool was used to assess the bias risk in RCTs by two reviewers (DY and JL). The assessment items included: (1) Allocation concealment (selection bias); (2) Random sequence generation (selection bias); (3) Blinding of outcome assessment (detection bias); Blinding of participants and personnel (performance bias); (4) Selective reporting (reporting bias); Incomplete outcome data (attrition bias); (5) Other bias. The assessment results would be classified into three categories, including high risk (H), low risk (L), and unclear risk (N). If two or more items were assessed as high risk, the trial would be considered as high risk. If all items of one trial were assessed as low risk or less than three items were of unclear risk, then the trial would be considered as low risk. The rest of the trials were classified as unclear risk [31].

### Other assessment and analysis

We evaluated the publication bias by funnel plots generated by Stata software. At the same time, we evaluated the robustness of each result by sensitivity analysis. In addition, we adopted the Grade approach to evaluate the certainty of evidence. Finally, we confirmed that patients were not involved in the design, implementation, reporting, or dissemination of this study.

## Results

### Literature study

**Fig 1:**
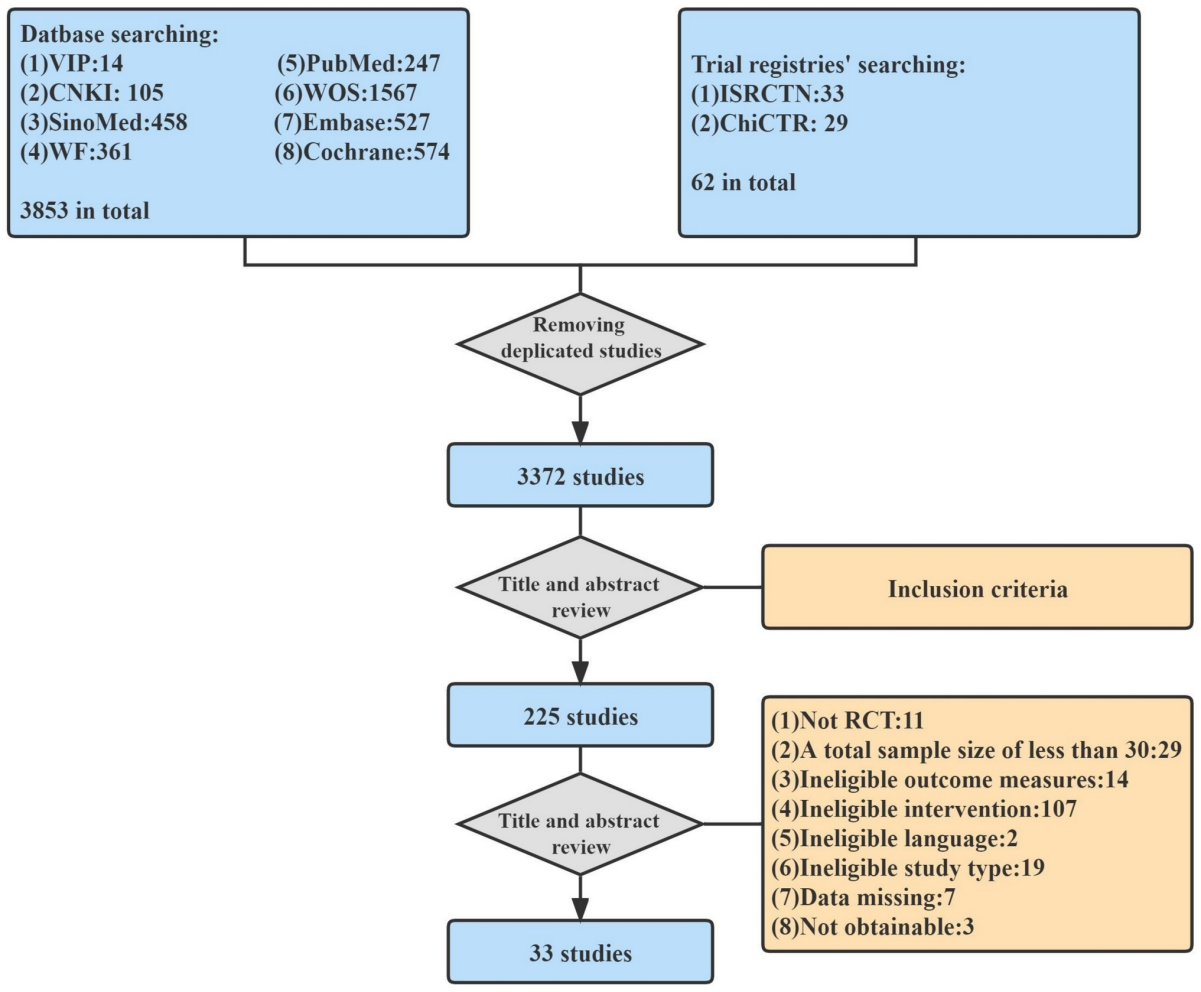
Flow chart of study selection.

In this study, we identified and screened titles and abstracts of 3915 studies from 8 databases and 2 clinical trial registries. After the duplication check and preliminary screening by two reviewers, a total of 225 studies meeting the basic requirements were reviewed in full text. Through further screening, 33 RCTs [29, 32–63] meeting all the inclusion criteria were final included in this study. The screening process is illustrated in Fig 1.

In the 33 RCTs included for further analysis, a total of 2246 subjects participated in the trials, of which 51 subjects withdrew for various reasons. Most studies clearly described the type of stroke (Cerebral Infarction or Cerebral Hemorrhage) in the baseline data [29, 32, 33, 35–42, 44–52, 54, 56–61] and the course of stroke [29, 33–44, 46–48, 50, 51–62]. Only 10 trials described the specific stroke stages [29, 36, 42, 50, 52] and Brunnstrom stage [32, 35, 46, 47, 59]. Most of trials provided an accurate description of the age of the subjects (mean age range: 45.10–75.64 years) as well as the gender ratio at the baseline period. Of all 33 trials, a total of 5 trials [29, 32, 37, 48, 61] had three intervention groups with a sample size ratio of 1:1:1, and the remaining studies had two intervention groups with a sample size ratio of 1:1. The details of interventions in included studies can be found in S2 Table.

We compared five different electrical stimulation methods in this study. At present, there is no uniform standard for the parameter setting of electric stimulation therapy, so the treatment dose, treatment frequency, treatment course and stimulation site of electric stimulation were not limited in the inclusion standard. The detailed parameter settings of different electrical stimulation methods are shown in S3 Table. Seven studies [33, 35, 41, 45–47, 52] did not record specific parameters, and most of studies had a dose range of 30–100 Hz for electrical stimulation. One study [50] increased the stimulation dose of NMES to 200 Hz, and another study [40] used a low stimulation dose of NMES at 1 Hz. The stimulation site in all studies was in the affected lower limb, except for four studies [32, 54, 58, 61] using tDCS in which the stimulation site was in the hemiplegic lower limb representative area of the motor cortex on the surface of the head, and one study [45] using TENS in which the stimulation site was on the fourth lumbar vertebra. The course of electrical stimulation in most studies was 20 mins or 30 mins, and the treatment frequency was mainly once a day, and 5 times per week.

### Risk of bias assessment

**Fig 2:**
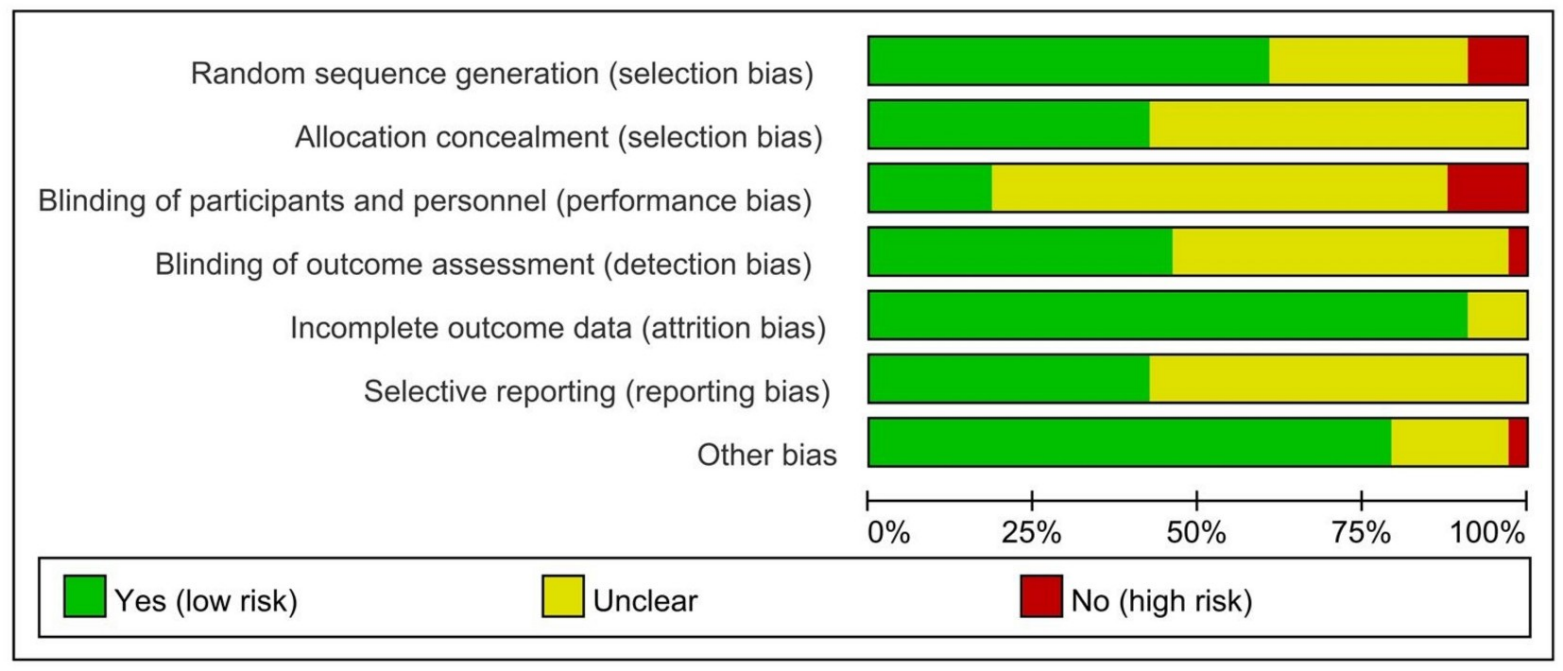
Risk of bias graph.

**Fig 3:**
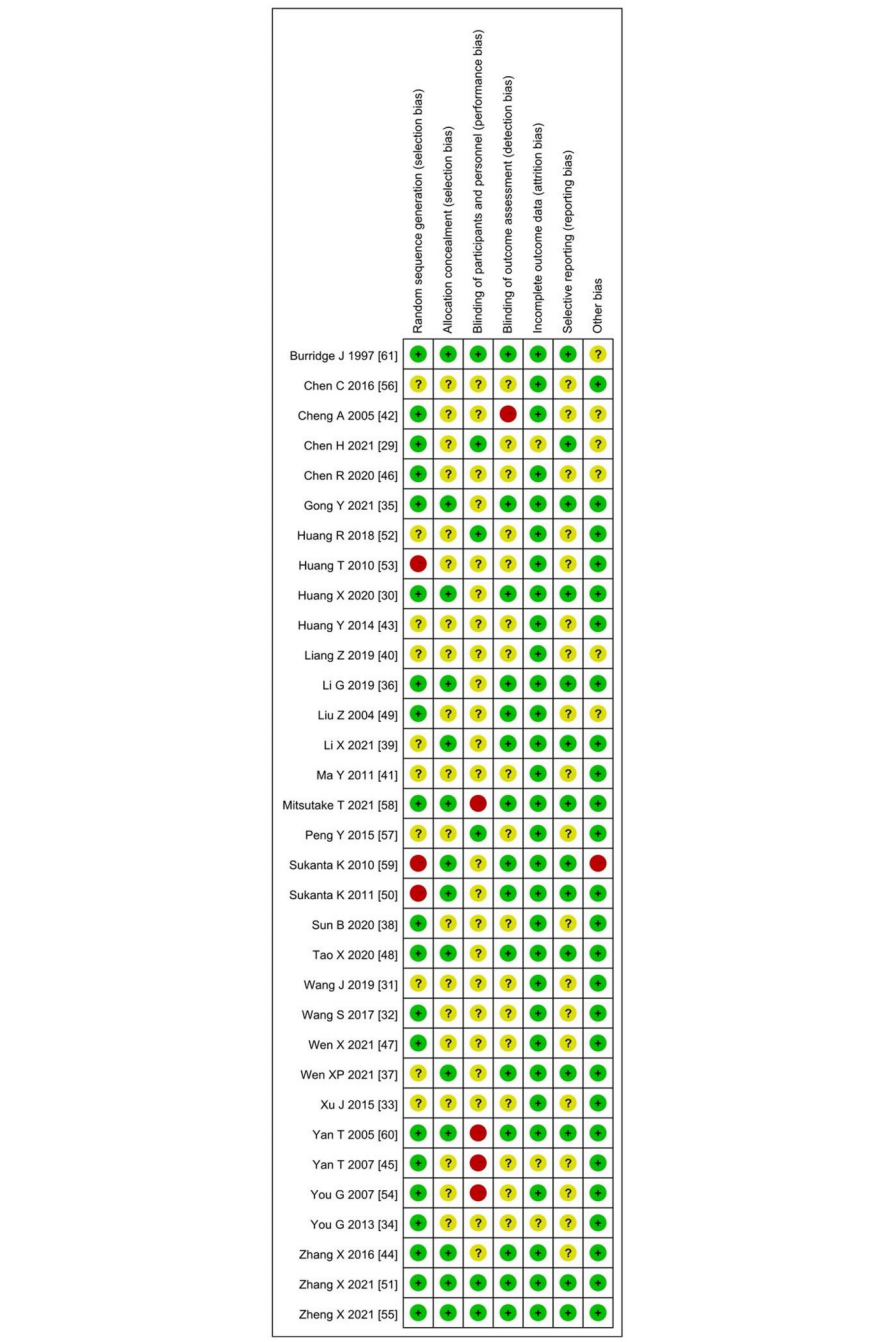
Risk of bias summary.

The results of bias risk assessment are depicted in Figs 2 and 3, and the detailed assessment results are presented in S4 Table. The results involved 9 studies that had high-risk items, 3 studies of which [53, 56, 62] did not describe random sequence generation methods, 4 studies of which [29, 48, 57, 61] were not blinded to subjects and study personnel, one study of which [62] was not blinded to the result evaluation and analysis personnel, and one study of which was considered otherwise biased due to missing baseline data. Moreover, it can be clearly seen that none of the high-risk bias trial was related to the following 3 assessment items: Allocation concealment (selection bias), Incomplete outcome data (attrition bias), and Selective reporting (reporting bias). In general, only one study [62] was classified as high-risk overall bias, accounting for approximately 3% of all studies. In addition, the results of the certainty of evidence (the Grade approach) are shown in S5 Table.

### Pairwise Meta-Analysis

The comparison results between two interventions using Pairwise Meta-Analysis are presented in S1 Fig. Meanwhile, the results of 5 outcome measures are summarized in Table 2 with the intervention groups having meaningful comprehensive effects highlighted in bold. We found that no matter what outcome measures was used, the treatment effect of FES, TENS, TEAS, and NMES combined with RT was superior to that of RT or RT combined with SS. When we used FMA-LE to evaluate the trial results, RT combined with TENS was less effective than RT combined with TEAS. Furthermore, the comparison results adopting BBS indicated that the treatment effect of RT combined with both FES and tDCS surpassed that of RT combined with both FES and SS on restoring the balance function.

**Table 2:** The results of five outcome measures.

Outcome Measure	Comparison	Number	SMD (95% CI)	I^2^ (%)	p
**FMA-LE**	RT+tDCS+FES vs RT+FES	1	0.20(-0.44,0.84)	-	-
	RT+tDCS+FES vs RT+tDCS	1	0.21(-0.43,0.86)	-	-
	RT+FES vs RT+tDCS	2	-0.75(-2.25,0.74)	94	<0.00001
	**RT+FES vs RT+SS**	1	**0.76(0.16,1.36)**	-	-
	**RT+FES vs RT**	11	**1.30(0.72,1.88)**	92	<0.00001
	**RT+TEAS vs RT+TENS**	1	**1.52(1.02,2.02)**	-	-
	RT+TEAS vs RT+SS	1	0.54(-0.09,1.16)	-	-
	**RT+TEAS vs RT**	4	**0.93(0.49,1.38)**	63	0.04
	**RT+TENS vs RT**	2	**1.01(0.63,1.39)**	0	0.39
	RT+SS vs RT	1	-0.01(-0.60,0.57)	-	-
	**RT+NMES vs RT**	4	**0.94(0.34,1.55)**	86	<0.00001
**BBS**	RT+tDCS+FES vs RT+FES	1	0.56(-0.09,1.21)	-	-
	RT+tDCS+FES vs RT+tDCS	1	0.18(-0.46,0.83)	-	-
	**RT+tDCS+FES vs RT+FES+SS**	1	**1.12(0.43,1.81)**	-	-
	RT+FES vs RT+tDCS	1	-0.35(-1.00,0.30)	-	-
	**RT+FES vs RT+SS**	1	**0.75(0.15,1.35)**	-	-
	**RT+FES vs RT**	7	**0.87(0.69,1.05)**	30	0.2
	**RT+TEAS vs RT+SS**	1	**0.79(0.15,1.42)**	-	-
	RT+SS vs RT	1	-0.01(-0.59,0.58)	-	-
	**RT+NMES vs RT**	1	**0.59(0.23,0.96)**	-	-
**MBI**	RT+tDCS+FES vs RT+FES	1	0.49(-0.16,1.13)	-	-
	RT+tDCS+FES vs RT+tDCS	1	0.37(-0.28,1.02)	-	-
	RT+FES vs RT+tDCS	2	-1.29(-3.62,1.03)	97	<0.00001
	**RT+FES vs RT**	5	**1.72(0.84,2.59)**	89	<0.00001
	**RT+TEAS vs RT**	2	**0.50(0.12,0.89)**	0	0.87
	**RT+NMES vs RT**	4	**2.04(0.58,3.51)**	97	<0.00001
**CSS**	RT+FES vs RT+SS	2	-0.51(-1.22,0.19)	54	0.14
	**RT+FES vs RT**	3	**-0.88(-1.28,-0.48)**	0	0.45
	**RT+TEAS vs RT+SS**	2	**-0.79(-1.26,-0.31)**	0	0.37
	RT+TEAS vs RT	1	-0.49(-1.18,0.21)	-	-
	RT+SS vs RT	3	-0.16(-0.54,0.23)	0	0.73
**10mMWS**	RT+tDCS+FES vs RT+tDCS	1	0.00(-0.84,0.84)	-	-
	RT+tDCS+FES vs RT+FES+SS	2	0.01(-0.50,0.51)	0	0.63
	**RT+FES vs RT**	5	**1.28(0.32,2.23)**	93	<0.00001
	RT+tDCS vs RT+FES+SS	1	0.20(-0.62,1.02)	-	-
	**RT+TENS vs RT**	1	**0.68(0.16,1.20)**	-	-

**Notes:** The bold values indicates a statistical difference.

### Network meta-analysis

**Fig 4:**
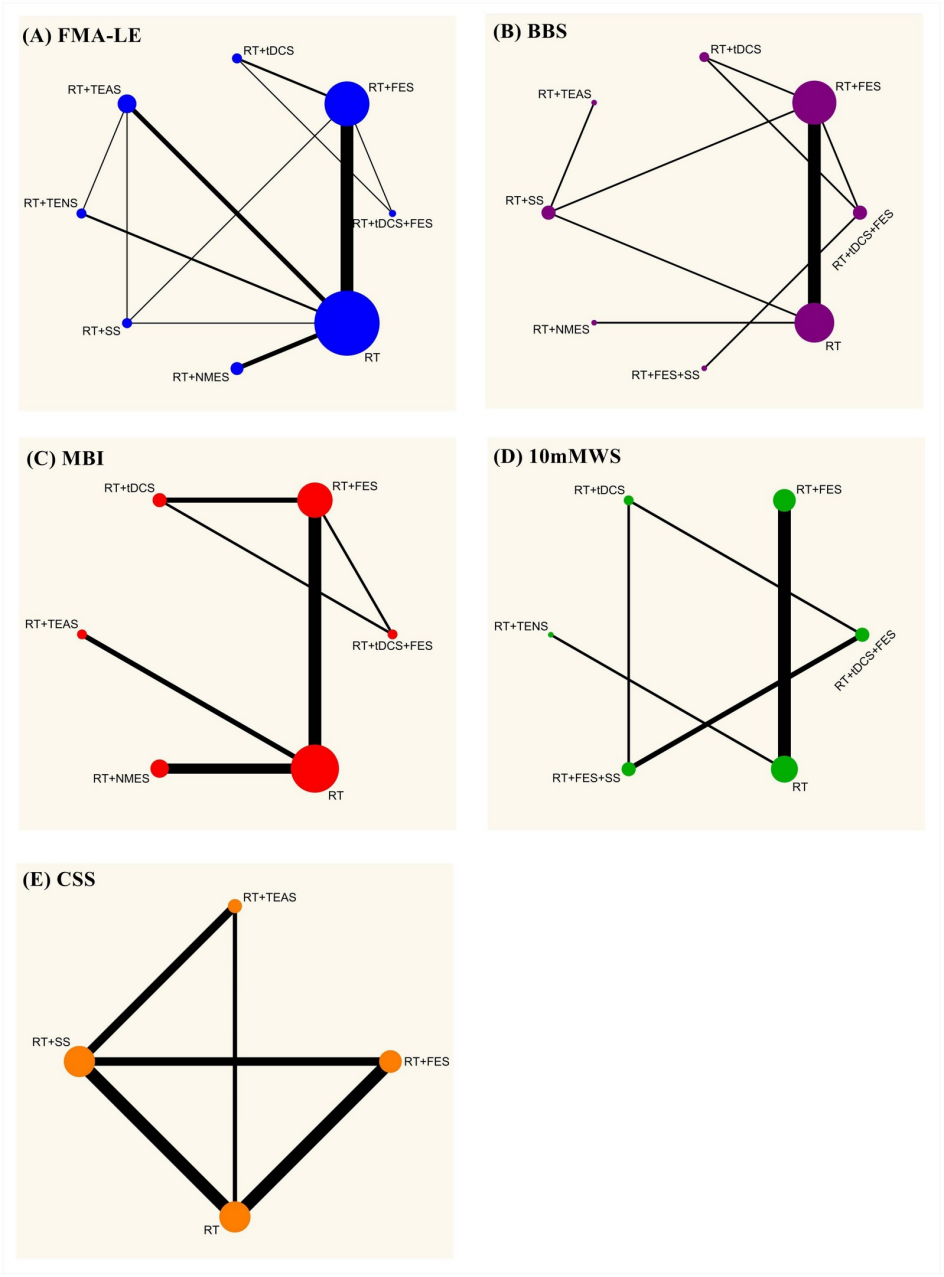
Network plot of outcomes. (A) FMA-LE, (B) BBS, (C) MBI, (D) 10mMWS, and (E) CSS.

Fig 4(A)–(4E) depict the network structures of five outcome measures (FMA-LE, BBS, MBI,10mMWS, CSS).

A total of 25 studies [32–42, 44–47, 49, 51–57, 59, 60] involving 1815 subjects used the FMA-LE; BBS was the measure for 11 trials [32, 35, 37, 39, 41, 42, 47, 56–58, 60] with 784 patients; MBI was adopted to 13 trials [32, 35, 37, 39, 41, 42, 47, 56–58, 60] with a total of 878 patients; 10mMWS was the outcome measure for 8 trials [35, 39, 43, 44, 58, 61, 62, 63] involving 466 patients; CSS was the outcome measure used for 5 trials [29, 37, 48, 57, 60] with 237 patients. As shown in Fig 4(A) and 4(B), the network structures among the interventions evaluated by FMA-LE and BBS were similar, while FMA-LE established one more association between TEAS, NMES, and FES. The results indicated that FMA-LE and BBS were the most frequently-used outcome measures for the evaluation of 8 interventions. As can be seen in Fig 4, no matter what outcome measure was used, the number of trials comparing RT with FES+RT was the largest. The CSS (shown in Fig 4(E)) only involved the comparison of 4 interventions and only covered two electrical stimulation methods (FES and TEAS). Additionally, there was no complete association established in the network plot of 10 mMWS (shown in Fig 4(D)), and there was no direct or indirect association between the two sets of interventions ((RT+FES, RT+TENS, RT) and (RT+tDCS, RT+FES+SS, RT+tDCS+FES)). Therefore, the network of the two sets of interventions were analyzed separately when performing the NMA analysis.

The NMA results of the inconsistency test showed that the direct or indirect comparison of each segment node was not statistically significant(*P*>0.05), further indicating that there was no evidence of design inconsistency. The convergence of the model was verified under the condition that the potential scale reduction factor was 1 (shown in S6 Table).

**Fig 5:**
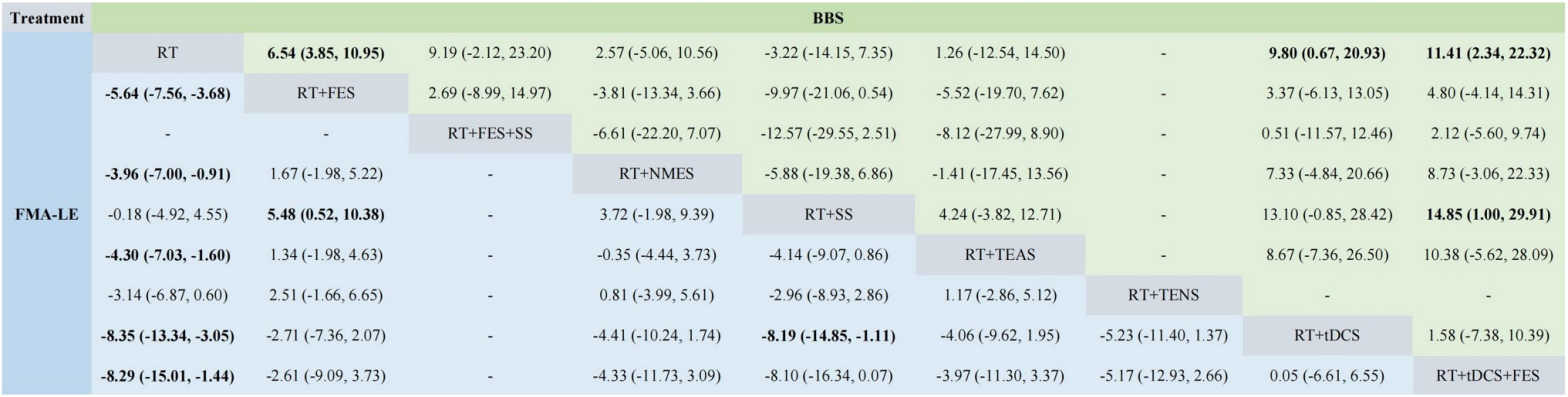
Network meta-analysis results for FMA-LE and BBS. Notes: The bold values indicates a statistical difference.

**Fig 6:**
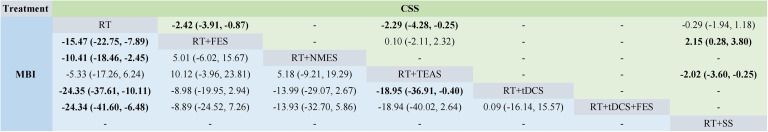
Network meta-analysis results for MBI and CSS. Notes: The bold values indicates a statistical difference.

**Fig 7:**
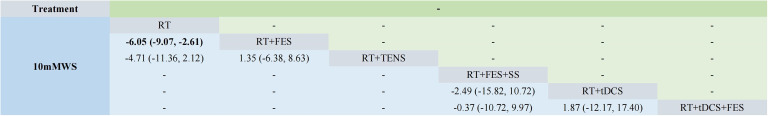
Network meta-analysis results for 10mMWS. Notes: The bold values indicates a statistical difference.

Figs 5–7 show the NMA results, and the intervention groups with significant combined effects are highlighted in bold. As illustrated in Fig 5 (FMA-LE and BBS), the treatment effects of FES, NMES, TEAS, tDCS, and tDCS+FES combined with RT were superior to that of RT in terms of improving motor function of the subjects’ lower limbs. Meanwhile, the treatment effect of FES+ tDCS was better than that of SS. FES, tDCS, and tDCS+FES combined with RT outperformed RT in improving the patients’ balance. When it comes to improving MBI, the treatment effects of FES, NMES, tDCS, tDCS+FES combined with RT were more desirable than that of RT, and the treatment effect of tDCS was significantly excellent than that of TEAS (shown in Fig 6). Moreover, the treatment effect of the combination of FES and TEAS with RT was better than that of RT in relieving patients’ lower limb spasticity (CSS). It can be seen from Fig 7 (10mMWS) that only the combination of FES and RT improved walking ability better than RT, while no remarkable difference has been shown in the treatment effect among other interventions.

In this study, we used a consistent model and adopted ADDIS to comprehensively rank the various interventions included in NMA. The ranking value for each intervention indicated its probability. As shown in S2 Fig, tDCS+FES combined with RT was most effective both in terms of improving FMA-LE scores, BBS and MBI, followed by tDCS and FES. FES performed best in relieving lower limb spasticity. Since the six interventions involving 10mMWS did not form a complete network structure, the two independent links in the network were ranked separately. The results indicated that based on RT, FES was superior to RT+TENS in improving patients’ walking ability, whereas tDCS outperformed FES+SS and tDCS+FES. The SUCRA scores are presented in S7 Table.

### Sensitivity analysis

After excluding the studies with high-risk bias and the studies with sample sizes less than 40 or drop-out rate more than 15%, we carried out sensitivity analysis of all pairwise meta-analyses, and the results remained unchanged. When we excluded the studies with drop-out rate exceeding 15%, the results of sensitivity analysis showed that the treatment effect of FES+RT was better than that of tDCS+RT in improving FMA-UE(SMD 0.03, 95%CI [-0.61,0.67]) and MBI(SMD -0.10, 95%CI [-0.74,0.55]).

### Publication bias

**Fig 8:**
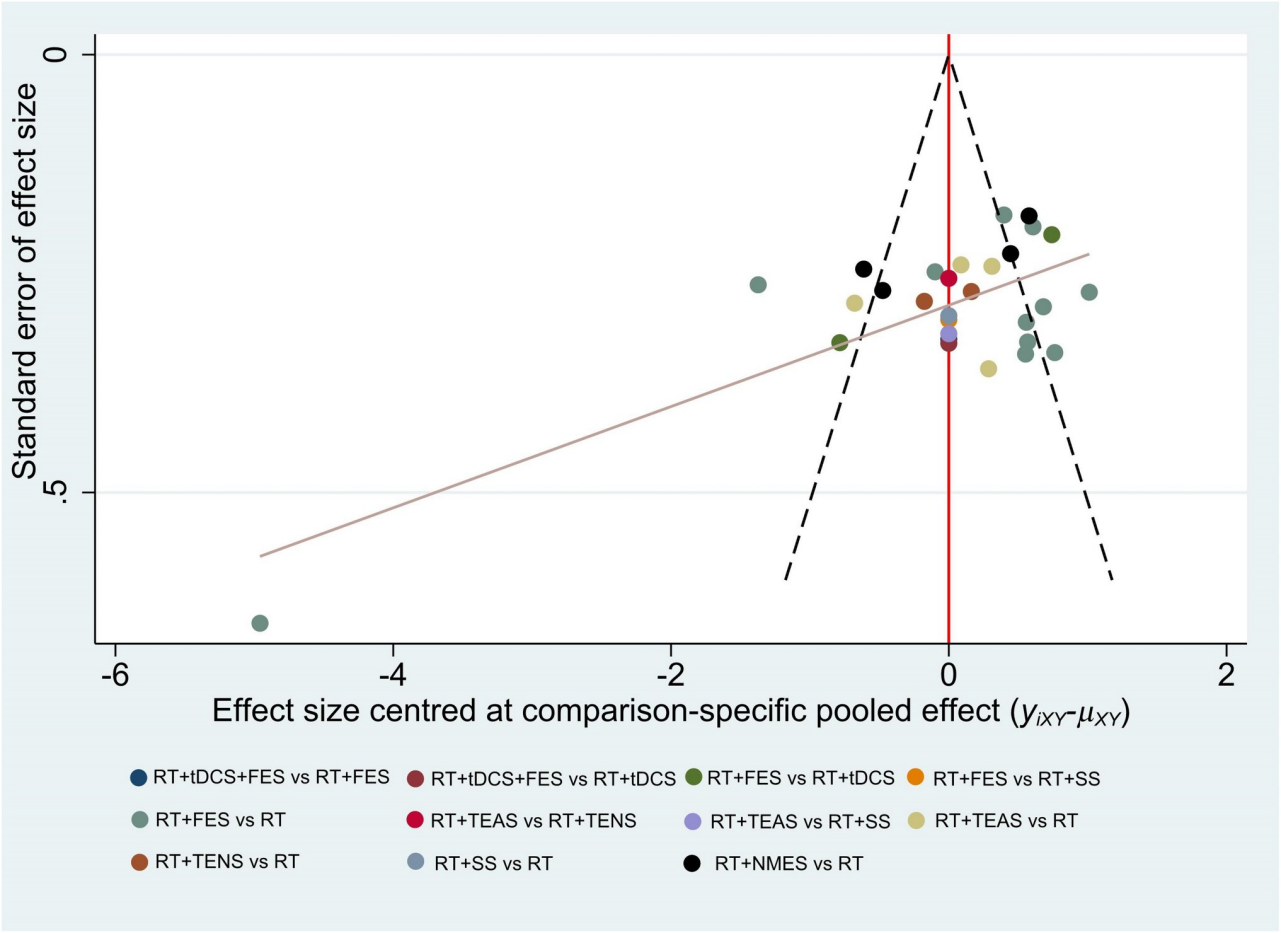
Funnel plot for the network meta-analysis of reduction in FMA-LE.

**Fig 9:**
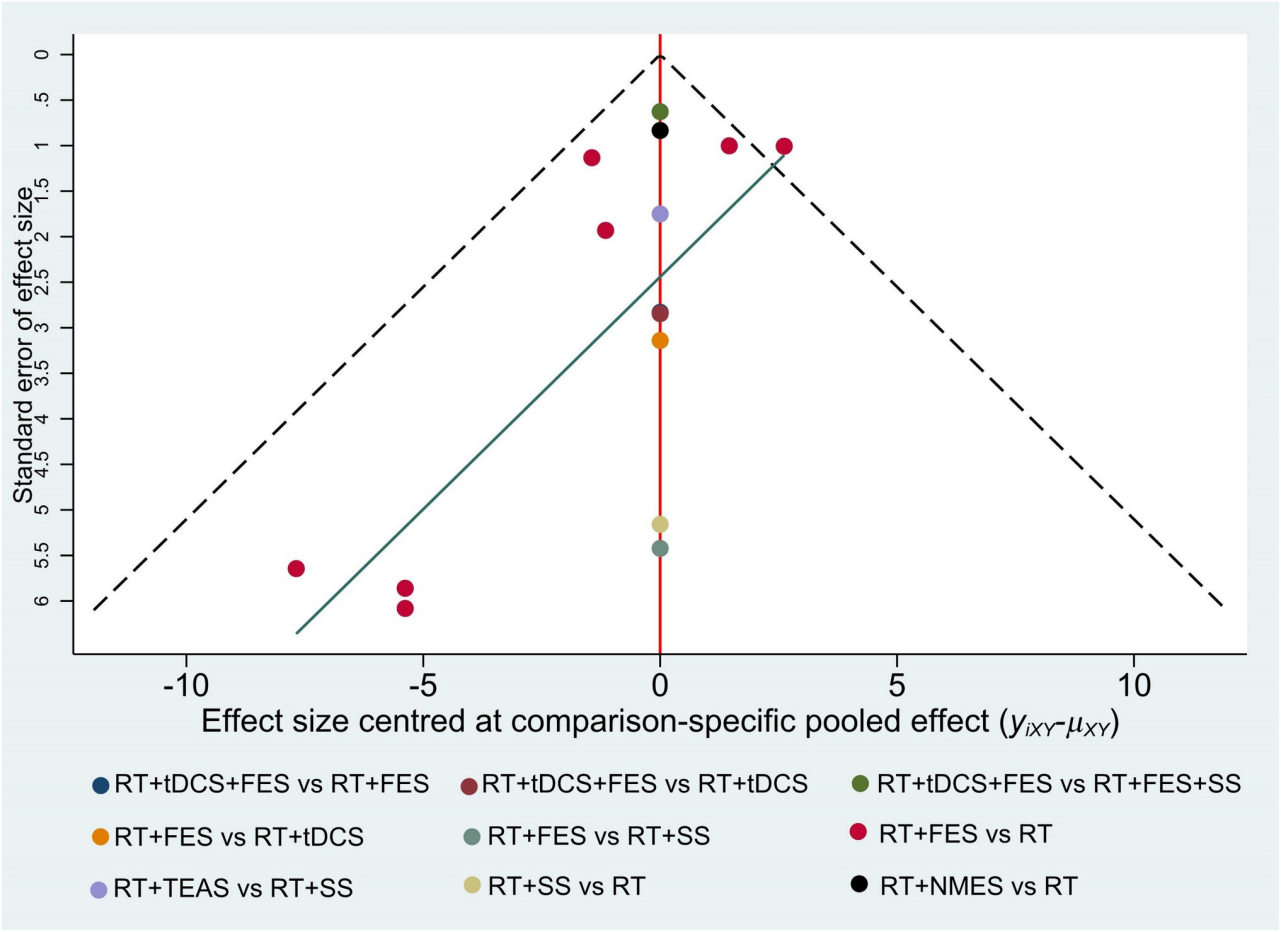
Funnel plot for the network meta-analysis of reduction in BBS.

**Fig 10:**
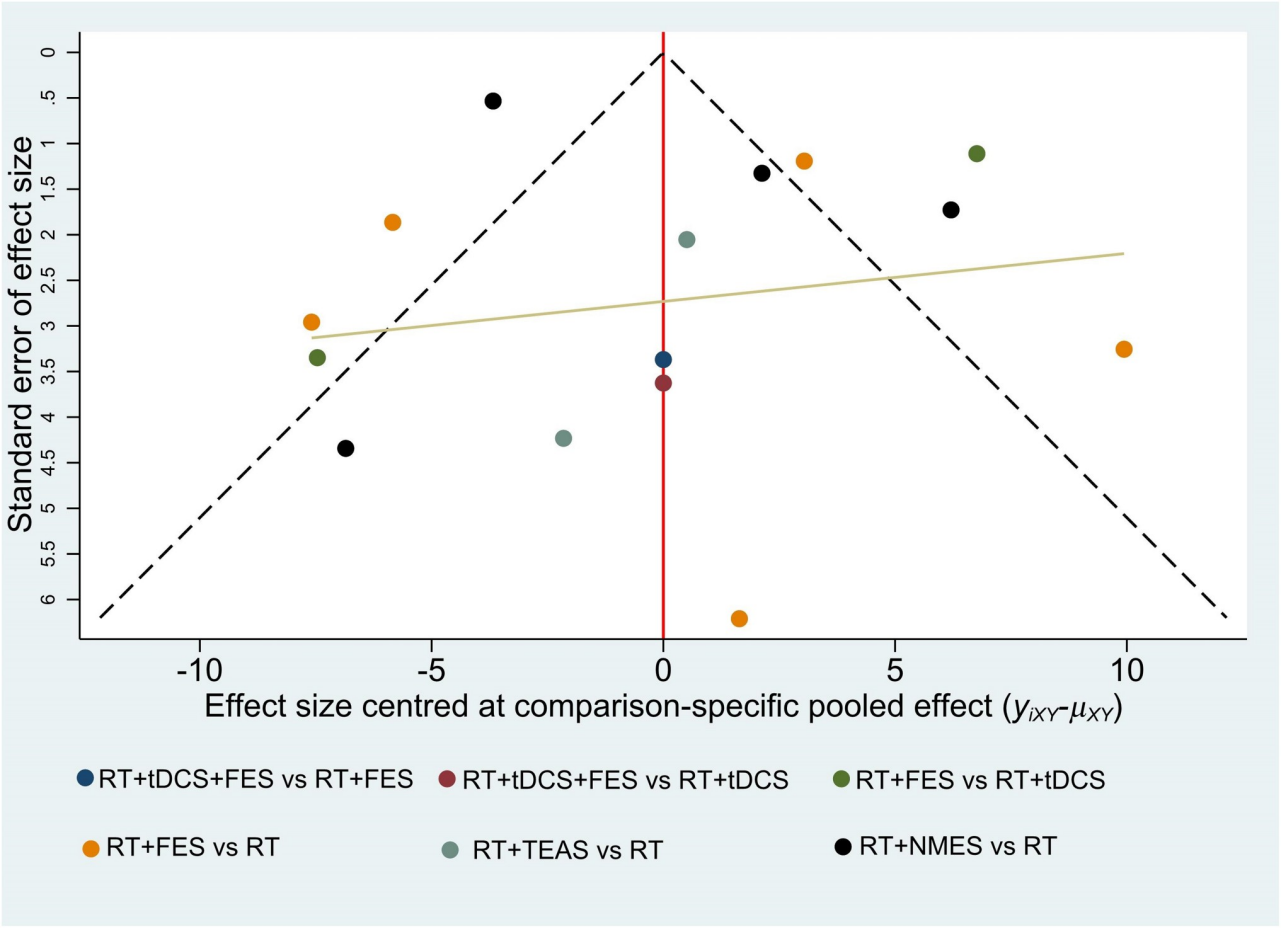
Funnel plot for the network meta-analysis of reduction in MBI.

We employed funnel plots to evaluate the publication bias. Owing to the small number of included studies, only CSS and 10mMWS were not evaluated by using funnel plots. As shown in these figures (Figs 8–10), most of the studies were distributed in the funnel (95% confidence interval). There were a small number of studies out of the 95% confidence interval, demonstrating that the potential heterogeneity did exist in these studies. (Figs 8 and 10). Due to the limited sample size of the included RCTs, most studies were distributed in the lower-middle part of the funnel plot. In addition, the missing angle of BBS (Fig 9) on the left side of the red vertical line (odds ratio = 0) may be relevant to the unpublished studies with negative results.

### Adverse events

In this paper, a total of 4 RCTs had adverse events. Among these RCTs, 3 RCTs [29, 48, 63] had adverse events of patient recurrent stroke, involving 5 patients and 4 interventions (RT, RT+SS, RT+FES, RT+TEAS). In one trial [29], one patient had gastric bleeding after using RT+FES. In another RCT [51], one patient had right lower limb thrombosis using RT alone.

## Discussion

### Main findings

In this study, we comprehensively searched studies from 8 databases and 2 clinical trial registries. Then, we performed a Pairwise Meta-Analysis and NMA on the 33 RCTs included in this study. Five frequently-used electrical stimulation methods for patients with limb dysfunction after stroke were selected in the analysis, including FES, TENS, TEAS, NMES, and tDCS. This is the first systematic review and NMA on different electrical stimulation methods in the treatment of lower limb dysfunction after stroke.

The NMA results demonstrated that the electrical stimulation methods combined with RT were more effective than RT. It can be indicated that electrical stimulation methods were capable of improving motor function of the lower limbs. Nevertheless, the treatment effect varied with different electrical stimulation methods. It can be deduced from the results that tDCS combined with FES was more effective in improving FMA-LE, BBS as well as MBI. Meanwhile, the treatment effect of tDCS or FES alone was second only to that of tDCS+FES, implying that these two electrical stimulation methods were optimal in treating lower limb dysfunction after stroke, and their combination can achieve more desirable results. FES is an electrical stimulation method by activating skeletal muscle with a constant frequency stimulation sequence [64], which has been developed as a popular treatment for lower limb dysfunction. FES has gradually been developed from a treatment method using electrophysiologically assisted devices to a treatment procedure that was capable of improving muscle control and residual motor nerve function [65]. tDCS is the only one of the five electrical stimulation methods with the stimulation site in the head. tDCS is a non-invasive neuromodulation technique [66], and its mechanism is promoting adaptive neuroplasticity [67]. Some researchers claimed that tDCS was beneficial for the recovery of motor function of patients with acute, subacute, or chronic stroke [68] under the premise of ensuring safety [69]. However, recent evidence indicated that tDCS cannot make any difference to the leg function, muscle strength, and cognitive function of patients after stroke [70]. In conclusion, although tDCS combined with RT showed significant advantages, the treatment effect of tDCS still needed to be further validated in large sample clinical controlled trials, considering the strict limitations of the interventions in this study.

In this paper, except for the stimulation areas of TENS and TEAS, the interventions of TENS and TEAS were almost the same. TENS stimulated the skin in the area of motor dysfunction, while TEAS stimulated acupuncture points. The previous studies have shown [71, 72] that TENS was beneficial to improving spasticity, muscle strength and gait capacity of stroke patients by regulating the spasticity based on various mechanisms, such as by increasing presynaptic inhibition or decreasing the excitability of the stretch reflex [73]. The results of NMA and FMA-LE ranking (S2 Fig) indicated that the treatment effect of TEAS was better than that of TENS, implying that the combined use of acupuncture treatment can improve the efficacy of electrical stimulation. Although the specific mechanism has not been found yet, the exploration of the effect of combined use of other electrical stimulation methods based on meridian-acupoint theory would be a very promising research direction. Furthermore, NMES was superior to TEAS in improving both FMA-LE and MBI scores, but inferior to TEAS in improving balance function (shown in S2 Fig). Some researchers believed that the actual clinical effectiveness of NMES depended on the systematic treatment scheme [74]. Additionally, the limitations of NMES for the recovery of motor function may be related to the recruitment of motor units during stimulation [75, 76]. It is worth noting that TEAS was not as good as SS in reducing CSS scores, which may result from the bias caused by only including one relevant study [48] (shown in Fig 6).

### Overall quality of evidence

In this study, 33 RCTs were included through careful selection, with a total of 2246 patients participating in the trial among these RCTs. Among these trials, only 4 trials reported adverse events, and the main adverse event was recurrent stroke, involving a total of seven patients and two electrical stimulation methods. However, there was no evidence that the adverse events were induced by electrical stimulation. Generally, electrical stimulation methods were safe. The appropriate population and the specific side effects still need to be explored in long-term clinical practice and high-quality clinical trials.

Due to the specificity of the electrical stimulation method, it is hard to realize double blindness in the intervention process. Unlike the drug RCTs, the lack of blindness is an inherent limitation of non-pharmacological studies involving interventions with physiotherapy methods [77] and is also considered as one of the main limitations of this study. We found that most of the trials missed assessment items after being evaluated through the Cochrane Collaboration risk of bias tool. The uncertain risk of bias may lead to insufficient effectiveness [78]. Furthermore, due to the limitation of the number of studies, the Grade approach may not be able to fully reflect the quality of evidence. Moreover, we evaluated the publication bias by funnel plots (shown in Figs 8–10), while we did not evaluate CSS and 10mMWS due to limitations in the number of trials. The horizontal lines of the funnel plots for the three outcome measures (depicted in Figs 8–10) were skewed, indicating the publication bias. On the one hand, the asymmetric funnel plot may be related to unpublished negative results [79, 80]. On the other hand, this result may also cause by some ongoing trials with unpublished data. Meanwhile, if some trial data can hardly be determined to be normal distribution, the quartile and the median or the minimum/maximum of the median would not be converted into the mean and standard deviation, thus affecting the publication bias to some degree. Base on the above reasons, we carried out the sensitivity analyses, and the results showed that most of the results were reliable.

### Strengths and limitations

The strengths of the present study can be summarized as follows. First, we conducted a comprehensive search and selection on 8 databases and 2 clinical trial registries according to PRISMA-NMA [25] and PRISMA guidelines and checklist [81]. Moreover, the rigorous data analysis approaches adopted in this study including NMA and Pairwise Meta-Analysis ensured the reliability of the final results. Secondly, this is a continuation study in which the methodology was extended and improved from the previous study [20]. In addition, this was the first academic research to compare and investigate the treatment effect of different electrical stimulation methods combined with RT for lower limb dysfunction after stroke.

Nevertheless, the limitations of this study can be concluded as follows. First of all, it can be seen from the baseline data of RCTs included, the course of some patients was significantly different. The same interventions may have different treatment effect on the stroke patients at different stage, thus influencing the comparison results of various electrical stimulation schemes. Secondly, the 5 three-arm trials [29, 32, 37, 48, 61] were divided into three comparison groups to compare different electrical stimulation schemes, but the sample size of each group was small, which may increase the risk of bias in this study. Finally, the differences in parameter settings may make a difference in the final treatment effect, so we sorted out the parameter details of various electrical stimulation methods in our preliminary work (S3 Table) and described the parameter details in results part Since there is still no parameter standard for different electrical stimulation methods on different diseases, this issue cannot be resolved temporarily.

## Conclusions

Compared with the method only using RT, the comprehensive treatment schemes combined with electrical stimulation methods presented remarkable superiority in the treatment of lower limb dysfunction after stroke. Meanwhile, the relatively high comprehensive ranking of TDCS or FES could provide new ideas for clinical treatment. Moreover, TEAS combined with acupuncture points exhibited greater treatment potential than conventional TENS. The results of this study provided a basis for further application of electrical stimulation methods. Owing to the limitation of quality and quantity of the included studies, high-quality RCTs are extremely need to offer powerful evidence to further support the results.

## Supporting information

S1 Table The PRISMA network meta-analysis checklist. (DOCX)

S2 Table Main characteristics of included RCTs. (DOCX)

S3 Table Details of electrical stimulation. (DOCX)

S4 Table Risk of bias assessment. (DOCX)

S5 Table The grade approach. (DOCX)

S6 Table Assessment of convergence of the model results. (DOCX)

S7 Table Treatment ranking and SUCRA for outcome data. (DOCX)

S1 Fig Pairwise Meta-Analysis. (DOCX)

S2 Fig Ranking probability. (DOCX)

S1 Checklist The PRISMA network meta-analysis checklist. (DOCX)
