# Supplementary material for: Optimization of electrical stimulation for the treatment of lower limb dysfunction after stroke: A systematic review and Bayesian network meta-analysis of randomized controlled trials
Source: PLoS One. 2023 May 11;18(5):e0285523. doi: 10.1371/journal.pone.0285523 (PMC10174537; doi:10.1371/journal.pone.0285523)
Supplement: S2 Table — (DOCX) [file pone.0285523.s002.docx]

**S2 Table.** Main characteristics of included RCTs.

| **Author**  **year** | **Country** | **Type of stroke(CH:Cerebral Infarction;CI:Cerebral Hemorrhage)** | **Stroke stage** | **Brunnstrom** | **Course of disease(days)** | | | **Sample size(Fall off quantities)** | **Allocation ratio** | **Age** | **Gender (M:F)** | **(A)** | **(B)** | **(C)** | **Duration of treatment(days)** | **Outcome Measure** | **Main Results** |
| --- | --- | --- | --- | --- | --- | --- | --- | --- | --- | --- | --- | --- | --- | --- | --- | --- | --- |
|  |  |  |  |  | **Treatment Group(A)** | **Control Group I(B)** | **Control Group II(C)** |  |  |  |  | **Treatment Group(A)** | **Control Group I(B)** | **Control Group II(C)** |  |  |  |
| Chen H 2021 | China | A:(CH:CI=11:8) B=(CH:CI=13:5) C=(CH:CI=8:7) | - | Ⅱ-Ⅳ | - | - | - | 66(10) | 1:1:1 | A:48.4±13.2  B:44.4±12.8  C:50.1±11.5 | A:(13:6)  B:(14:5)  C:(14:4) | RT+tDCS+FES | RT+FES | RT+tDCS | 12 | 1.FMA-LE  2.BBS  3.MBI | 1.A=B=C  2.A＞B  3.A＞C |
| Huang X 2020 | China | A:(CH:CI=18:20) B=(CH:CI=16:22) | - | - | 76.54±16.82 | 74.22±17.31 | - | 76 | 1:1 | A:69.37±7.80  B:68.54±7.12 | A:(30:8)  B:(28:10) | RT+TEAS | RT | - | 28 | 1.FMA-LE | 1.A＞B |
| Wang J 2019 | China | - | - | - | 46.03±8.86 | 43.20±10.47 | - | 80 | 1:1 | A:47.67±11.63  B:45.10±10.49 | A:(22:18)  B:(25:15) | RT+TEAS | RT+TENS | - | 28 | 1.FMA-LE | 1.A＞B |
| Wang S  2017 | China | CH:CI=43:89 | - | ＞Ⅱ | 49.8±12.3 | 49.4±11.9 | - | 132 | 1:1 | A:63.4±14.5  B:62.0±11.8 | A:(31:35)  B:(30:36) | RT+FES | RT | - | 28 | 1.FMA-LE  2.BBS  3.10mMWS | 1.A＞B  2.A＞B  3.A＞B |
| Xu J  2015 | China | A:(CH:CI=2:38) B=(CH:CI=2:38) | Acute stage | - | 0.5-8 | 0.5-8 | - | 80 | 1:1 | A:65.6±12.7  B:63.1±10.1 | A:(27:13)  B:(24:16) | RT+NMES | RT | - | 14 | 1.FMA-LE  2.MBI | 1.A＞B  2.A＞B |
| You G  2013 | China | A:(CH:CI=5:18) B=(CH:CI=4:19) C=(CH:CI=5:17) | - | - | 24.6±20.2 | 23.7±16.9 | 25.2±19.4 | 76(8) | 1:1:1 | A:62.7±10.9  B:61.6±9.7  C:64.2±8.8 | A:(13:10)  B:(14:9)  C:(15:7) | RT+FES | RT+SS | RT | 21 | 1.FMA-LE  2.BBS  3.CSS | 1.A＞B=C  2.A＞B=C  3.A＞B=C |
| Gong Y 2021 | China | A:(CH:CI=5:29) B=(CH:CI=8:25) | - | - | 127.8±100.8 | 164.4±114 | - | 70(3) | 1:1 | 62.58±8.87 | A:(17:17)  B:(16:17) | RT+TEAS | RT | - | 28 | 1.FMA-LE  2.MBI | 1.A＞B  2.A＞B |
| Li G  2019 | China | A:(CH:CI=12:18) B=(CH:CI=11:19) | - | - | 13.07±8.13 | 12.67±7.56 | - | 60 | 1:1 | A:58.53±7.13  B:58.40±7.02 | A:(17:13)  B:(16:14) | RT+FES | RT | - | 14 | 1.FMA-LE  2.BBS  3.10mMWS | 1.A＞B  2.A＞B  3.A＞B |
| Wen XP  2021 | China | A:(CH:CI=25:19) B=(CH:CI=24:20) | - | - | 23.1±3.5 | 25.2±5.6 | - | 88 | 1:1 | A:65.3±3.2  B:66.1±3.7 | A:(24:20)  B:(26:18) | RT+NMES | RT | - | 28 | 1.FMA-LE  2.MBI | 1.A＞B  2.A＞B |
| Sun B  2020 | China | A:(CH:CI=20:21) B=(CH:CI=16:25) | - | - | 66.3±11.7 | 67.8±8.1 | - | 82 | 1:1 | A:55.94±8.38  B:56.02±8.22 | A:(22:19)  B:(24:17) | RT+FES | RT | - | - | 1.FMA-LE  2.BBS  3.MBI | 1.A＞B  2.A＞B  3.A＞B |
| Li X  2021 | China | A:(CH:CI=42:18)  B=(CH:CI=49:11) | Sequelae period | - | ＜90 | | - | 120 | 1:1 | A:56.26±9.36  B:59.21±9.25 | A:(32:28)  B:(34:26) | RT+NMES | RT | - | 28 | 1.FMA-LE  2.BBS | 1.A＞B  2.A＞B |
| Liang Z 2019 | China | - | - | - | 25.71±3.25 | 25.61±3.21 | - | 80 | 1:1 | A:57.81±1.41  B:57.21±1.25 | A:(20:20)  B:(22:18) | RT+FES | RT | - | 15 | 1.MBI  2.10mMWS | 1.A＞B  2.A＞B |
| Ma Y  2011 | China | CH:CI=22:38 | - | - | 103.56±22.48 | | - | 60 | 1:1 | 53.80±13.04 | (39:21) | RT+TENS | RT | - | 32 | 1.FMA-LE  2.10mMWS | 1.A＞B  2.A＞B |
| Cheng A  2005 | China | CH:CI=20:40 | - | - | - | - | - | 60 | 1:1 | 58±5 | (38:22) | RT+TENS | RT | - | 28 | 1.FMA-LE | 1.A＞B |
| Huang Y 2014 | China | A:(CH:CI=7:21)  B=(CH:CI=5:22) | - | ≥Ⅲ | 50.76±18.23 | 51.13±17.59 | - | 55 | 1:1 | A:58.25±7.64  B:57.63±9.19 | A:(15:13)  B:(14:13) | RT+FES | RT | - | 35 | 1.FMA-LE | 1.A＞B |
| Zhang X 2016 | China | A:(CH:CI=12:43)  B=(CH:CI=10:45) | - | ＜Ⅱ | 56.7±20.1 | 57.9±24.3 | - | 110 | 1:1 | A:63.86±16.81  B:62.94±17.23 | A:(29:26)  B:(28:27) | RT+FES | RT | - | 28 | 1.FMA-LE  2.BBS | 1.A＞B  2.A＞B |
| Yan T  2007 | China | A:(CH:CI=3:14)  B=(CH:CI=3:14)  C=(CH:CI=3:13) | - | - | 9.2±4.4 | 9.9±2.6 | 8.7±3.3 | 56(6) | 1:1:1 | A:68.4±9.6  B:72.8±7.4  C:70.4±7.6 | A:(8:9)  B:(9:8)  C:(8:8) | RT+TEAS | RT+SS | RT | 21 | 1.CSS | 1.A＞B |
| Chen R 2020 | China | CI | - | - | - | - | - | 70 | 1:1 | A:60.2±2.1  B:59.8±1.9 | A:(20:15)  B:(21:14) | RT+NMES | RT | - | 42 | 1.FMA-LE  2.MBI | 1.A＞B  2.A＞B |
| Wen X  2021 | China | CI | Acute stage | - | 3.08±0.72 | 3.11±0.15 | - | 82 | 1:1 | A:67.26±2.11  B:69.97±3.16 | A:(24:17)  B:(26:15) | RT+NMES | RT | - | 15 | 1.MBI | 1.A＞B |
| Tao X  2020 | China | A:(CH:CI=10:26)  B=(CH:CI=11:25) | - | - | 41.64±18.04 | 42.75±17.29 | - | 73(1) | 1:1 | A:57.61±9.42  B:58.47±8.96 | A:(24:12)  B:(23:13) | RT+TEAS | RT | - | 28 | 1.FMA-LE  2.MBI | 1.A＞B  2.A＞B |
| Liu Z  2004 | China | A:(CH:CI=16:40)  B=(CH:CI=19:37) | Acute stage | - | - | - | - | 112 | 1:1 | A:60.7±13.8  B:61.3±12.5 | A:(33:23)  B:(31:25) | RT+FES | RT | - | 15 | 1.FMA-LE | 1.A＞B |
| Sukanta K 2011 | India | - | - | - | 519±564 | 546±354 | - | 51 | 1:1 | A:49.1±8.8  B:50.1±10.4 | A:(4:12)  B:(2:12) | RT+FES | RT | - | 84 | 1.FMA-LE | 1.A＞B |
| Zhang X 2021 | China | A:(CH:CI=19:42)  B=(CH:CI=22:39) | - | - | 43.45±5.66 | 42.45±4.75 | - | 122 | 1:1 | A:58.18±11.70  B:56.11±12.01 | A:(43:18)  B:(38:23) | RT+FES | RT+tDCS | - | 56 | 1.FMA-LE  2.MBI | 1.A＜B  2.A＜B |
| Huang R 2018 | China | - | - | - | 12.2±6.5 | 9.8±5.7 | - | 36 | 1:1 | A:58.2±11.7  B:58.6±11.3 | A:(9:8)  B:(7:11) | RT+FES | RT | - | 21 | 1.FMA-LE  2.MBI | 1.A＞B  2.A＞B |
| Huang T 2010 | China | A:(CH:CI=3:17)  B=(CH:CI=2:18) | - | - | 12.0±8.5 | 13.4±7.6 | - | 40 | 1:1 | A:73.8±10.8  B:70.3±9.9 | A:(16:4)  B:(17:3) | RT+FES | RT | - | 21 | 1.FMA-LE  2.BBS  3.MBI | 1.A＞B  2.A＞B  3.A＞B |
| You G  2007 | China | A:(CH:CI=2:17)  B=(CH:CI=2:16) | - | - | 25.9±21.3 | 22.7±16.6 | - | 44(7) | 1:1 | A:60.8±10.8  B:64.1±9.7 | A:(11:8)  B:(10:8) | RT+FES | RT | - | 21 | 1.FMA-LE  2.BBS  3.CSS  4.MBI | 1.A＞B  2.A＞B  3.A＞B  4.A＞B |
| Zheng X 2021 | China | A:(CH:CI=11:9)  B=(CH:CI=10:8) | - | - | 80±11 | 79±10 | - | 38 | 1:1 | A:55.54±10.57  B:57.84±11.32 | A:(13:7)  B:(12:6) | RT+FES+tDCS | RT+FES+SS | - | 14 | 1.BBS  2.10mMWS | 1.A＞B  2.A＜B |
| Chen C 2016 | China | A:(CH:CI=4:12)  B=(CH:CI=6:11) | - | Ⅰ-Ⅴ | 65.44±29.25 | 67.76±30.87 | - | 33 | 1:1 | A:59.38±9.59  B:60.59±10.75 | A:(14:2)  B:(13:4) | RT+TEAS | RT | - | 28 | 1.FMA-LE  2.MBI | 1.A＞B  2.A＞B |
| Peng Y  2015 | China | A:(CH:CI=6:15)  B=(CH:CI=7:13) | - | - | 61.5±25.5 | 55.5±34.5 | - | 48(7) | 1:1 | A:65.4±12.8  B:68.8±10.6 | A:(12:9)  B:(11:9) | RT+TEAS | RT+SS | - | 21 | 1.FMA-LE  2.BBS  3.CSS | 1.A＞B  2.A＞B  3.A＞B |
| Mitsutake T 2021 | Japan | A:(CH:CI=3:9)  B=(CH:CI=1:10)  C=(CH:CI=2:9) | - | - | 37.08±27.34 | 34.64±17.76 | 44.64±31.73 | 37(3) | 1:1:1 | A:67.33±12.09  B:75.64±10.97  C:74.91±9.19 | A:(9:3)  B:(4:7)  C:(6:5) | RT+FES+SS | RT+tDCS | RT+FES+tDCS | 7 | 1.10mMWS | 1.A=B=C |
| Sukanta K 2010 | India | - | - | - | 600±? | 450±? | - | 30 | 1:1 | A:49.5±8.9  B:47.1±12.4 | A:(12:4)  B:(12:2) | RT+FES | RT | - | 84 | 1.10mMWS | 1.A＞B |
| Yan T  2005 | China | A:(CH:CI=2:11)  B=(CH:CI=2:13)  C=(CH:CI=2:11) | Acute stage | - | 8.7±5.8 | 10.1±2.8 | 9.1±3.5 | 46(5) | 1:1:1 | A:68.2±7.7  B:73.3±8.1  C:70.4±7.6 | A:(7:6)  B:(7:8)  C:(6:7) | RT+FES | RT+SS | RT | 21 | 1.CSS | 1.A＞B |
| Burridge J 1997 | Britain | - | - | - | - | - | - | 33(1) | 1:1 | A:52.3±14.3  B:61.3±8.6 | A:(10:6)  B:(13:3) | RT+FES | RT | - | 30 | 1.10mMWS | 1.A＞B |
