# Supplementary material for: Optimization of electrical stimulation for the treatment of lower limb dysfunction after stroke: A systematic review and Bayesian network meta-analysis of randomized controlled trials
Source: PLoS One. 2023 May 11;18(5):e0285523. doi: 10.1371/journal.pone.0285523 (PMC10174537; doi:10.1371/journal.pone.0285523)
Supplement: S3 Table — (DOCX) [file pone.0285523.s003.docx]

**S3 Table.** Details of electrical stimulation.

| **Author year** | **Frequency(Hz)/**  **intensity(mA) of electrical stimulation** | **The site of electrical stimulation** | **Duration of electrical stimulation** | **Number of treatments** | **Frequency of treatment(weeks/days)** |
| --- | --- | --- | --- | --- | --- |
| Chen H 2021 | tDCS:2mA  FES:30Hz | FES:Motor points of tibialis anterior muscle,quadriceps femoris,gastrocnemius muscle and biceps femoris  tDCS:The anode was placed in the representative area of primary motor cortex of hemiplegic lower limbs (M1 area)，The cathode was placed at the superior margin of the contralateral orbit | 20min | RT+tDCS+FES:19  RT+FES:19  RT+tDCS:18 | 1'/D |
| Huang X 2020 | - | Acupuncture points:Taichong;Qiuxu | 20min | RT+TEAS:38  RT:38 | 1'/D |
| Wang J 2019 | TEAS:50Hz  TENS:50 Hz | TEAS:Zusanli,Yanglingquan,Feiyang,Kunlun  TENS:Zusanli,Yanglingquan | 10min | RT+TEAS:40  RT+TENS:40 | 6'/W;1'/D |
| Wang S 2017 | - | Tibialis anterior muscle | 30min | RT+FES:66  RT:66 | 5-6'/W;1'/D |
| Xu J 2015 | 30Hz;15-40mA | Motor points of tibialis anterior muscle,peroneal longus and brevis | 30min | RT+NMES:40  RT:40 | 1'/D |
| You G 2013 | 30Hz | Motor points of tibialis anterior muscle,peroneal longus and brevis | 30min | RT+FES:23  RT+SS:23  RT:22 | 1'/D |
| Gong Y 2021 | 50Hz | Acupuncture points:Zusanli,Shangjuxu,Xiajuxu,jiexi,Yinlingquan | 20min | RT+TEAS:34  RT:33 | 6'/W;1'/D |
| Li G  2019 | 30Hz | Tibialis anterior muscle,quadriceps femoris,gastrocnemius muscle and hamstring | 30min | RT+FES:30  RT:30 | 6'/W;1'/D |
| Wen X 2021 | 1Hz | Tibialis anterior muscle and rectus femoris | 30min | RT+NMES:44  RT:44 | 6'/W;1'/D |
| Sun B  2020 | - | Diseased lower limbs | 20min | RT+FES:41  RT:41 | 1'/D |
| Li X  2021 | 50Hz;20-30mA | Muscle surface of bilateral lower limbs | 30min | RT+NMES:60  RT:60 | 1'/D |
| Liang Z 2019 | 30Hz | Motor points of tibialis anterior muscle,peroneal longus and brevis | 30min | RT+FES:40  RT:40 | 1'/D |
| Ma Y 2011 | 100Hz | Common peroneal nerve | 30min | RT+TENS:30  RT:30 | 1'/D |
| Cheng A 2005 | - | The anode is placed on the lumbar(L4),the cathode is placed at the fibular head and the palm of foot | - | RT+TENS:30  RT:30 | 6'/W;1'/D |
| Huang Y 2014 | - | Common peroneal nerve and tibialis anterior muscle | - | RT+FES:28  RT:27 | 5'/W |
| Zhang X 2016 | - | Motor points of tibialis anterior muscle and common peroneal nerve | 15-20min | RT+FES:55  RT:55 | 6'/W;2'/D |
| Yan T  2007 | 100Hz | Acupuncture points:Zusanli,jiexi,Yanglingquan,Kunlun | 60min | RT+TEAS:17  RT+SS:17  RT:16 | 5'/W;1'/D |
| Chen R 2020 | 28mA | Gluteus medius,sartorius muscle,tibialis anterior muscle,peroneal muscle | 30min | RT+NMES:35  RT:35 | 1'/D |
| Wen X 2021 | 200Hz | Flexor and extensor | 20min | RT+NMES:41  RT:41 | 1'/D |
| Tao X  2020 | 100Hz | Acupuncture points:Zusanli,jiexi,Yanglingquan,Sanyinjiao | 30min | RT+TEAS:36  RT:36 | 5'/W;1'/D |
| Liu Z  2004 | - | Motor points of tibialis anterior muscle | 30min | RT+FES:56  RT:56 | 2'/D |
| Sukanta K 2011 | 35Hz | the tibialis anterior muscle of the paretic limb | 20-30min | RT+FES:27  RT:24 | 5'/W |
| Zhang X 2021 | FES:15-50Hz,4-20mA  tDCS:2mA | FES:the erector spine, gluteus maximus, quadriceps femoris, hamstring, tibialis anterior, and gastrocnemius muscles of the affected side;tDCS:The anode was placed on the stimulation site in the central anterior motor area of the brain, and the cathode was placed on the contralateral forehead | 20min | RT+FES:61  RT+tDCS:61 | 5'/W |
| Huang R 2018 | 30Hz | Motor points of tibialis anterior muscle,quadriceps femoris,gastrocnemius muscle and biceps femoris | 30min | RT+FES:18  RT:18 | 5'/W;1'/D |
| Huang T 2010 | 30Hz | Motor points of tibialis anterior muscle,peroneal longus and brevis | 30min | RT+FES:20  RT:20 | 5'/W;1'/D |
| You G  2007 | 30Hz | Motor points of tibialis anterior muscle,peroneal longus and brevis | 30min | RT+FES:19  RT:18 | 5'/W;1'/D |
| Zheng X 2021 | tDCS:2mA  FES:30Hz | tDCS:The anode was placed in the representative area of primary motor cortex of hemiplegic lower limbs (M1 area),the cathode was placed in the prefrontal region of the contralateral brain  FES:Motor points of tibialis anterior muscle,peroneal longus and brevis | 20min | RT+FES+tDCS:20  RT+FES+SS:18 | 5'/W;1'/D |
| Chen C 2016 | 100Hz | Acupuncture points:Zusanli,Yanglingquan | 30min | RT+TEAS:16  RT:17 | 5'/W |
| Peng Y 2015 | 100Hz | Acupuncture points:Zusanli,Taichong,Yanglingquan,Kunlun | 60min | RT+TEAS:21  RT+SS:20 | 5'/W;1'/D |
| Mitsutake T 2021 | FES:33Hz  tDCS:2mA | tDCS:Electrodes were positioned with the anode over the presumed leg region of the injured hemisphere, just lateral to the Cz position of the international electroencephalogram 10-20 system, and the cathode over the contralateral supraorbital region. | 20min | RT+FES+SS:12  RT+tDCS:11  RT+FES+tDCS:11 | 1'/D |
| Sukanta K 2010 | 40Hz | Diseased lower limbs | 30min | RT+FES:16  RT:14 | 5'/W;1'/D |
| Yan T  2014 | 30Hz | quadriceps,hamstring,tibialis anterior (TA), and medial gastrocnemius (MG) | 30min | RT+FES:13  RT+SS:15  RT:13 | 5'/W;1'/D |
| Burridge J 1997 | 40Hz | Diseased foots | - | RT+FES:16  RT:16 | 1'/D |
