# Supplementary material for: Optimization of electrical stimulation for the treatment of lower limb dysfunction after stroke: A systematic review and Bayesian network meta-analysis of randomized controlled trials
Source: PLoS One. 2023 May 11;18(5):e0285523. doi: 10.1371/journal.pone.0285523 (PMC10174537; doi:10.1371/journal.pone.0285523)
Supplement: S4 Table — (DOCX) [file pone.0285523.s004.docx]

**S4 Table.** Risk of Bias Assessment.

| **Author year** | **Random sequence generation (selection bias)** | **Allocation concealment (selection bias)** | **Blinding of participants and personnel (performance bias)** | **Blinding of outcome assessment (detection bias)** | **Incomplete outcome data (attrition bias)** | **Selective reporting (reporting bias)** | **Other bias** |
| --- | --- | --- | --- | --- | --- | --- | --- |
| Burridge J 1997 | **L** | **L** | **L** | **L** | **L** | **L** | **N** |
| Chen C 2016 | **N** | **N** | **N** | **N** | **L** | **N** | **L** |
| Chen H 2021 | **L** | **N** | **L** | **N** | **N** | **L** | **N** |
| Chen R 2020 | **L** | **N** | **N** | **N** | **L** | **N** | **N** |
| Cheng A 2005 | **L** | **N** | **N** | **H** | **L** | **N** | **N** |
| Gong Y 2021 | **L** | **L** | **N** | **L** | **L** | **L** | **L** |
| Huang R 2018 | **N** | **N** | **L** | **N** | **L** | **N** | **L** |
| Huang T 2010 | **H** | **N** | **N** | **N** | **L** | **N** | **L** |
| Huang X 2020 | **L** | **L** | **N** | **L** | **L** | **L** | **L** |
| Huang Y 2014 | **N** | **N** | **N** | **N** | **L** | **N** | **L** |
| Li G 2019 | **L** | **L** | **N** | **L** | **L** | **L** | **L** |
| Li X 2021 | **N** | **L** | **N** | **L** | **L** | **L** | **L** |
| Liang Z 2019 | **N** | **N** | **N** | **N** | **L** | **N** | **N** |
| Liu Z 2004 | **L** | **N** | **N** | **L** | **L** | **N** | **N** |
| Ma Y 2011 | **N** | **N** | **N** | **N** | **L** | **N** | **L** |
| Mitsutake T 2021 | **L** | **L** | **H** | **L** | **L** | **L** | **L** |
| Peng Y 2015 | **N** | **N** | **L** | **N** | **L** | **N** | **L** |
| Sukanta K 2010 | **H** | **L** | **N** | **L** | **L** | **L** | **H** |
| Sukanta K 2011 | **H** | **L** | **N** | **L** | **L** | **L** | **L** |
| Sun B 2020 | **L** | **N** | **N** | **N** | **L** | **N** | **L** |
| Tao X 2020 | **L** | **L** | **N** | **L** | **L** | **L** | **L** |
| Wang J 2019 | **N** | **N** | **N** | **N** | **L** | **N** | **L** |
| Wang S 2017 | **L** | **N** | **N** | **N** | **L** | **N** | **L** |
| Wen X 2021 | **L** | **N** | **N** | **N** | **L** | **N** | **L** |
| Wen XP 2021 | **N** | **L** | **N** | **L** | **L** | **L** | **L** |
| Xu J 2015 | **N** | **N** | **N** | **N** | **L** | **N** | **L** |
| Yan T 2007 | **L** | **N** | **H** | **N** | **N** | **N** | **L** |
| Yan T 2005 | **L** | **L** | **H** | **L** | **L** | **L** | **L** |
| You G 2007 | **L** | **N** | **H** | **N** | **L** | **N** | **L** |
| You G 2013 | **L** | **N** | **N** | **N** | **N** | **N** | **L** |
| Zhang X 2016 | **L** | **L** | **N** | **L** | **L** | **N** | **L** |
| Zhang X 2021 | **L** | **L** | **L** | **L** | **L** | **L** | **L** |
| Zheng X 2021 | **L** | **L** | **L** | **L** | **L** | **L** | **L** |
