# Supplementary material for: Optimization of electrical stimulation for the treatment of lower limb dysfunction after stroke: A systematic review and Bayesian network meta-analysis of randomized controlled trials
Source: PLoS One. 2023 May 11;18(5):e0285523. doi: 10.1371/journal.pone.0285523 (PMC10174537; doi:10.1371/journal.pone.0285523)
Supplement: S5 Table — (DOCX) [file pone.0285523.s005.docx]

**S5 Table.** The Grade approach.

**Question:** Should RT+tDCS+FES vs RT+FES be used for lower Limb dysfunction after stroke?

| **Quality assessment** | | | | | | | **No of patients** | | **Effect** | | **Quality** | **Importance** |
| --- | --- | --- | --- | --- | --- | --- | --- | --- | --- | --- | --- | --- |
|  |  |  |  |  |  |  |  |  |  |  |  |  |
| **No of studies** | **Design** | **Risk of bias** | **Inconsistency** | **Indirectness** | **Imprecision** | **Other considerations** | **RT+tDCS+FES** | **RT+FES** | **Relative**  **(95% CI)** | **Absolute** |  |  |
| **FMA-LE (Better indicated by higher values)** | | | | | | | | | | | | |
| 1 | randomised trials | no serious risk of bias | no serious inconsistency | no serious indirectness | serious^1^ | reporting bias^2^ | 19 | 19 | - | SMD 0.20 higher (0.44 lower to 0.84 higher) | ⊕⊕OO  LOW | CRITICAL |
| **BBS (Better indicated by higher values)** | | | | | | | | | | | | |
| 1 | randomised trials | no serious risk of bias | no serious inconsistency | no serious indirectness | serious^1^ | reporting bias^2^ | 19 | 19 | - | SMD 0.56 higher (0.09 lower to 1.21 higher) | ⊕⊕OO  LOW | IMPORTANT |
| **MBI (Better indicated by higher values)** | | | | | | | | | | | | |
| 1 | randomised trials | no serious risk of bias | no serious inconsistency | no serious indirectness | serious^1^ | reporting bias^2^ | 19 | 19 | - | SMD 0.49 higher (0.16 lower to 1.13 higher) | ⊕⊕OO  LOW | IMPORTANT |

^1^ The sample size was small

^2^ Funnel plot can not be judged, and there was no related interest statement

**Question:** Should RT+tDCS+FES vs RT+tDCS be used for lower Limb dysfunction after stroke?

| **Quality assessment** | | | | | | | **No of patients** | | **Effect** | | **Quality** | **Importance** |
| --- | --- | --- | --- | --- | --- | --- | --- | --- | --- | --- | --- | --- |
|  |  |  |  |  |  |  |  |  |  |  |  |  |
| **No of studies** | **Design** | **Risk of bias** | **Inconsistency** | **Indirectness** | **Imprecision** | **Other considerations** | **RT+tDCS+FES** | **RT+tDCS** | **Relative**  **(95% CI)** | **Absolute** |  |  |
| **FMA-LE (Better indicated by higher values)** | | | | | | | | | | | | |
| 1 | randomised trials | no serious risk of bias | no serious inconsistency | no serious indirectness | serious^1^ | reporting bias^2^ | 19 | 18 | - | SMD 0.21 higher (0.43 lower to 0.86 higher) | ⊕⊕OO  LOW | CRITICAL |
| **BBS (Better indicated by higher values)** | | | | | | | | | | | | |
| 1 | randomised trials | no serious risk of bias | no serious inconsistency | no serious indirectness | serious^1^ | reporting bias^2^ | 19 | 18 | - | SMD 0.18 higher (0.46 lower to 0.83 higher) | ⊕⊕OO  LOW | IMPORTANT |
| **MBI (Better indicated by higher values)** | | | | | | | | | | | | |
| 1 | randomised trials | no serious risk of bias | no serious inconsistency | no serious indirectness | serious^1^ | reporting bias^2^ | 19 | 18 | - | SMD 0.37 higher (0.28 lower to 1.02 higher) | ⊕⊕OO  LOW | IMPORTANT |
| **10mMWS (Better indicated by higher values)** | | | | | | | | | | | | |
| 1 | randomised trials | serious^3^ | no serious inconsistency | no serious indirectness | serious^1^ | none | 11 | 11 | - | SMD 0.00 higher (0.84 lower to 0.84 higher) | ⊕⊕OO  LOW | IMPORTANT |

^1^ The sample size was small

^2^ Funnel plot can not be judged, and there was no related interest statement

^3^ Subjects and study personnel were not blinded

**Question:** Should RT+FES vs RT+tDCS be used for lower Limb dysfunction after stroke?

| **Quality assessment** | | | | | | | **No of patients** | | **Effect** | | **Quality** | **Importance** |
| --- | --- | --- | --- | --- | --- | --- | --- | --- | --- | --- | --- | --- |
|  |  |  |  |  |  |  |  |  |  |  |  |  |
| **No of studies** | **Design** | **Risk of bias** | **Inconsistency** | **Indirectness** | **Imprecision** | **Other considerations** | **RT+FES** | **RT+tDCS** | **Relative**  **(95% CI)** | **Absolute** |  |  |
| **FMA-LE (Better indicated by higher values)** | | | | | | | | | | | | |
| 2 | randomised trials | no serious risk of bias | serious^1^ | no serious indirectness | no serious imprecision | none | 80 | 79 | - | SMD 0.75 lower (2.25 lower to 0.74 higher) | ⊕⊕⊕O  MODERATE | CRITICAL |
| **BBS (Better indicated by higher values)** | | | | | | | | | | | | |
| 1 | randomised trials | no serious risk of bias | no serious inconsistency | no serious indirectness | serious^2^ | reporting bias^3^ | 19 | 18 | - | SMD 0.35 lower (1 lower to 30 higher) | ⊕⊕OO  LOW | IMPORTANT |
| **MBI (Better indicated by higher values)** | | | | | | | | | | | | |
| 2 | randomised trials | no serious risk of bias | serious^1^ | no serious indirectness | no serious imprecision | none | 80 | 79 | - | SMD 1.29 lower (3.62 lower to 1.03 higher) | ⊕⊕⊕O  MODERATE | IMPORTANT |

^1^ Heterogeneity is high (I² > 50%).

^2^ The sample size was small

^3^ Funnel plot can not be judged, and there was no related interest statement

**Question:** Should RT+FES vs RT+SS be used for lower Limb dysfunction after stroke?

| **Quality assessment** | | | | | | | **No of patients** | | **Effect** | | **Quality** | **Importance** |
| --- | --- | --- | --- | --- | --- | --- | --- | --- | --- | --- | --- | --- |
|  |  |  |  |  |  |  |  |  |  |  |  |  |
| **No of studies** | **Design** | **Risk of bias** | **Inconsistency** | **Indirectness** | **Imprecision** | **Other considerations** | **RT+FES** | **RT+SS** | **Relative**  **(95% CI)** | **Absolute** |  |  |
| **FMA-LE (Better indicated by higher values)** | | | | | | | | | | | | |
| 1 | randomised trials | no serious risk of bias | no serious inconsistency | no serious indirectness | serious^1^ | reporting bias^2^ | 23 | 23 | - | SMD 0.76 higher (0.16 to 1.36 higher) | ⊕⊕OO  LOW | CRITICAL |
| **BBS (Better indicated by higher values)** | | | | | | | | | | | | |
| 1 | randomised trials | no serious risk of bias | no serious inconsistency | no serious indirectness | serious^1^ | reporting bias^2^ | 23 | 23 | - | SMD 0.75 higher (0.15 to 1.35 higher) | ⊕⊕OO  LOW | IMPORTANT |
| **CSS (Better indicated by lower values)** | | | | | | | | | | | | |
| 2 | randomised trials | no serious risk of bias | serious^3^ | no serious indirectness | no serious imprecision | none | 36 | 38 | - | SMD 0.51 lower (1.22 lower to 0.19 higher) | ⊕⊕⊕O  MODERATE | IMPORTANT |

^1^ The sample size was small

^2^ Funnel plot can not be judged, and there was no related interest statement

^3^ Heterogeneity is high (I² > 50%).

**Question:** Should RT+FES vs RT be used for lower Limb dysfunction after stroke?

| **Quality assessment** | | | | | | | **No of patients** | | **Effect** | | **Quality** | **Importance** |
| --- | --- | --- | --- | --- | --- | --- | --- | --- | --- | --- | --- | --- |
|  |  |  |  |  |  |  |  |  |  |  |  |  |
| **No of studies** | **Design** | **Risk of bias** | **Inconsistency** | **Indirectness** | **Imprecision** | **Other considerations** | **RT+FES** | **RT** | **Relative**  **(95% CI)** | **Absolute** |  |  |
| **FMA-LE (Better indicated by higher values)** | | | | | | | | | | | | |
| 11 | randomised trials | no serious risk of bias | serious^1^ | no serious indirectness | no serious imprecision | none | 383 | 377 | - | SMD 1.30 higher (72 to 1.88 higher) | ⊕⊕⊕O  MODERATE | CRITICAL |
| **BBS (Better indicated by higher values)** | | | | | | | | | | | | |
| 7 | randomised trials | no serious risk of bias | no serious inconsistency | no serious indirectness | no serious imprecision | none | 254 | 252 | - | SMD 0.87 higher (0.69 to 1.05 higher) | ⊕⊕⊕⊕  HIGH | IMPORTANT |
| **MBI (Better indicated by higher values)** | | | | | | | | | | | | |
| 5 | randomised trials | no serious risk of bias | serious^1^ | no serious indirectness | no serious imprecision | none | 138 | 137 | - | SMD 1.72 higher (0.84 to 2.59 higher) | ⊕⊕⊕O  MODERATE | IMPORTANT |
| **CSS (Better indicated by lower values)** | | | | | | | | | | | | |
| 3 | randomised trials | serious^2^ | no serious inconsistency | no serious indirectness | no serious imprecision | none | 55 | 53 | - | SMD 0.88 lower (1.28 to 0.48 lower) | ⊕⊕⊕O  MODERATE | IMPORTANT |
| **10mMWS (Better indicated by higher values)** | | | | | | | | | | | | |
| 5 | randomised trials | no serious risk of bias | serious^1^ | no serious indirectness | no serious imprecision | none | 168 | 166 | - | SMD 1.28 higher (0.32 to 2.23 higher) | ⊕⊕⊕O  MODERATE | IMPORTANT |

^1^ Heterogeneity is high (I² > 50%).

^2^ Subjects and study personnel were not blinded

**Question:** Should RT+TEAS vs RT+TENS be used for lower Limb dysfunction after stroke?

| **Quality assessment** | | | | | | | **No of patients** | | **Effect** | | **Quality** | **Importance** |
| --- | --- | --- | --- | --- | --- | --- | --- | --- | --- | --- | --- | --- |
|  |  |  |  |  |  |  |  |  |  |  |  |  |
| **No of studies** | **Design** | **Risk of bias** | **Inconsistency** | **Indirectness** | **Imprecision** | **Other considerations** | **RT+TEAS** | **RT+TENS** | **Relative**  **(95% CI)** | **Absolute** |  |  |
| **FMA-LE (Better indicated by higher values)** | | | | | | | | | | | | |
| 1 | randomised trials | no serious risk of bias | no serious inconsistency | no serious indirectness | no serious imprecision | reporting bias^1^ | 40 | 40 | - | SMD 1.52 higher (1.02 to 2.02 higher) | ⊕⊕⊕O  MODERATE | CRITICAL |

^1^ Funnel plot can not be judged, and there was no related interest statement

**Question:** Should RT+TEAS vs RT+SS be used for lower Limb dysfunction after stroke?

| **Quality assessment** | | | | | | | **No of patients** | | **Effect** | | **Quality** | **Importance** |
| --- | --- | --- | --- | --- | --- | --- | --- | --- | --- | --- | --- | --- |
|  |  |  |  |  |  |  |  |  |  |  |  |  |
| **No of studies** | **Design** | **Risk of bias** | **Inconsistency** | **Indirectness** | **Imprecision** | **Other considerations** | **RT+TEAS** | **RT+SS** | **Relative**  **(95% CI)** | **Absolute** |  |  |
| **FMA-LE (Better indicated by higher values)** | | | | | | | | | | | | |
| 1 | randomised trials | no serious risk of bias | no serious inconsistency | no serious indirectness | serious^1^ | reporting bias^2^ | 21 | 20 | - | SMD 0.54 higher (0.09 lower to 1.16 higher) | ⊕⊕OO  LOW | CRITICAL |
| **BBS (Better indicated by higher values)** | | | | | | | | | | | | |
| 1 | randomised trials | no serious risk of bias | no serious inconsistency | no serious indirectness | serious^1^ | reporting bias^2^ | 21 | 20 | - | SMD 0.79 higher (0.15 to 1.42 higher) | ⊕⊕OO  LOW | IMPORTANT |
| **CSS (Better indicated by lower values)** | | | | | | | | | | | | |
| 2 | randomised trials | no serious risk of bias | no serious inconsistency | no serious indirectness | serious^1^ | reporting bias^2^ | 38 | 37 | - | SMD 0.79 lower (1.26 to 0.31 lower) | ⊕⊕OO  LOW | IMPORTANT |

^1^ The sample size was small

^2^ Funnel plot can not be judged, and there was no related interest statement

**Question:** Should RT+TEAS vs RT be used for lower Limb dysfunction after stroke?

| **Quality assessment** | | | | | | | **No of patients** | | **Effect** | | **Quality** | **Importance** |
| --- | --- | --- | --- | --- | --- | --- | --- | --- | --- | --- | --- | --- |
|  |  |  |  |  |  |  |  |  |  |  |  |  |
| **No of studies** | **Design** | **Risk of bias** | **Inconsistency** | **Indirectness** | **Imprecision** | **Other considerations** | **RT+TEAS** | **RT** | **Relative**  **(95% CI)** | **Absolute** |  |  |
| **FMA-LE (Better indicated by higher values)** | | | | | | | | | | | | |
| 4 | randomised trials | no serious risk of bias | serious^1^ | no serious indirectness | no serious imprecision | none | 124 | 124 | - | SMD 0.93 higher (0.49 to 1.38 higher) | ⊕⊕⊕O  MODERATE | CRITICAL |
| **MBI (Better indicated by higher values)** | | | | | | | | | | | | |
| 2 | randomised trials | no serious risk of bias | no serious inconsistency | no serious indirectness | no serious imprecision | reporting bias^2^ | 52 | 53 | - | SMD 0.50 higher (0.12 to 0.89 higher) | ⊕⊕⊕O  MODERATE | IMPORTANT |
| **CSS (Better indicated by lower values)** | | | | | | | | | | | | |
| 1 | randomised trials | serious^3^ | no serious inconsistency | no serious indirectness | serious^4^ | reporting bias^2^ | 17 | 16 | - | SMD 0.49 lower (1.18 lower to 0.21 higher) | ⊕OOO  VERY LOW | IMPORTANT |

^1^ Heterogeneity is high (I² > 50%).

^2^ Funnel plot can not be judged, and there was no related interest statement

^3^ Subjects and study personnel were not blinded

^4^ The sample size was small

**Question:** Should RT+TENS vs RT be used for lower Limb dysfunction after stroke?

| **Quality assessment** | | | | | | | **No of patients** | | **Effect** | | **Quality** | **Importance** |
| --- | --- | --- | --- | --- | --- | --- | --- | --- | --- | --- | --- | --- |
|  |  |  |  |  |  |  |  |  |  |  |  |  |
| **No of studies** | **Design** | **Risk of bias** | **Inconsistency** | **Indirectness** | **Imprecision** | **Other considerations** | **RT+TENS** | **RT** | **Relative**  **(95% CI)** | **Absolute** |  |  |
| **FMA-LE (Better indicated by higher values)** | | | | | | | | | | | | |
| 2 | randomised trials | no serious risk of bias | no serious inconsistency | no serious indirectness | no serious imprecision | reporting bias^1^ | 60 | 60 | - | SMD 1.01 higher (0.63 to 1.39 higher) | ⊕⊕⊕O  MODERATE | CRITICAL |
| **10mMWS (Better indicated by higher values)** | | | | | | | | | | | | |
| 1 | randomised trials | no serious risk of bias | no serious inconsistency | no serious indirectness | no serious imprecision | reporting bias^1^ | 30 | 30 | - | SMD 0.68 higher (0.16 to 1.2 higher) | ⊕⊕⊕O  MODERATE | IMPORTANT |

^1^ Funnel plot can not be judged, and there was no related interest statement

**Question:** Should RT+SS vs RT be used for lower Limb dysfunction after stroke?

| **Quality assessment** | | | | | | | **No of patients** | | **Effect** | | **Quality** | **Importance** |
| --- | --- | --- | --- | --- | --- | --- | --- | --- | --- | --- | --- | --- |
|  |  |  |  |  |  |  |  |  |  |  |  |  |
| **No of studies** | **Design** | **Risk of bias** | **Inconsistency** | **Indirectness** | **Imprecision** | **Other considerations** | **RT+SS** | **RT** | **Relative**  **(95% CI)** | **Absolute** |  |  |
| **FMA-LE (Better indicated by higher values)** | | | | | | | | | | | | |
| 1 | randomised trials | no serious risk of bias | no serious inconsistency | no serious indirectness | serious^1^ | reporting bias^2^ | 23 | 22 | - | SMD 0.01 lower (0.6 lower to 0.57 higher) | ⊕⊕OO  LOW | CRITICAL |
| **BBS (Better indicated by higher values)** | | | | | | | | | | | | |
| 1 | randomised trials | no serious risk of bias | no serious inconsistency | no serious indirectness | serious^1^ | reporting bias^2^ | 23 | 22 | - | SMD 0.01 lower (0.59 lower to 0.58 higher) | ⊕⊕OO  LOW | IMPORTANT |
| **CSS (Better indicated by lower values)** | | | | | | | | | | | | |
| 3 | randomised trials | serious^3^ | no serious inconsistency | no serious indirectness | no serious imprecision | none | 55 | 51 | - | SMD 0.16 lower (0.54 lower to 0.23 higher) | ⊕⊕⊕O  MODERATE | IMPORTANT |

^1^ The sample size was small

^2^ Funnel plot can not be judged, and there was no related interest statement

^3^ Subjects and study personnel were not blinded

**Question:** Should RT+NMES vs RT be used for lower Limb dysfunction after stroke?

| **Quality assessment** | | | | | | | **No of patients** | | **Effect** | | **Quality** | **Importance** |
| --- | --- | --- | --- | --- | --- | --- | --- | --- | --- | --- | --- | --- |
|  |  |  |  |  |  |  |  |  |  |  |  |  |
| **No of studies** | **Design** | **Risk of bias** | **Inconsistency** | **Indirectness** | **Imprecision** | **Other considerations** | **RT+NMES** | **RT** | **Relative**  **(95% CI)** | **Absolute** |  |  |
| **FMA-LE (Better indicated by higher values)** | | | | | | | | | | | | |
| 4 | randomised trials | no serious risk of bias | serious^1^ | no serious indirectness | no serious imprecision | none | 179 | 179 | - | SMD 0.94 higher (0.34 to 1.55 higher) | ⊕⊕⊕O  MODERATE | CRITICAL |
| **BBS (Better indicated by higher values)** | | | | | | | | | | | | |
| 1 | randomised trials | no serious risk of bias | no serious inconsistency | no serious indirectness | no serious imprecision | reporting bias^2^ | 60 | 60 | - | SMD 0.59 higher (0.23 to 0.96 higher) | ⊕⊕⊕O  MODERATE | IMPORTANT |
| **MBI (Better indicated by higher values)** | | | | | | | | | | | | |
| 4 | randomised trials | no serious risk of bias | serious^1^ | no serious indirectness | no serious imprecision | reporting bias^2^ | 160 | 160 | - | SMD 2.04 higher (0.58 to 3.51 higher) | ⊕⊕OO  LOW | IMPORTANT |

^1^ Heterogeneity is high (I² > 50%).

^2^ Funnel plot can not be judged, and there was no related interest statement

**Question:** Should RT+tDCS+FES vs RT+FES+SS be used for lower Limb dysfunction after stroke?

| **Quality assessment** | | | | | | | **No of patients** | | **Effect** | | **Quality** | **Importance** |
| --- | --- | --- | --- | --- | --- | --- | --- | --- | --- | --- | --- | --- |
|  |  |  |  |  |  |  |  |  |  |  |  |  |
| **No of studies** | **Design** | **Risk of bias** | **Inconsistency** | **Indirectness** | **Imprecision** | **Other considerations** | **RT+tDCS+FES** | **RT+FES+SS** | **Relative**  **(95% CI)** | **Absolute** |  |  |
| **BBS (Better indicated by higher values)** | | | | | | | | | | | | |
| 1 | randomised trials | no serious risk of bias | no serious inconsistency | no serious indirectness | serious^1^ | reporting bias^2^ | 20 | 18 | - | SMD 1.12 higher (0.43 to 1.81 higher) | ⊕⊕OO  LOW | IMPORTANT |
| **10mMWS (Better indicated by higher values)** | | | | | | | | | | | | |
| 2 | randomised trials | no serious risk of bias | no serious inconsistency | no serious indirectness | no serious imprecision | none | 31 | 30 | - | SMD 0.01 higher (0.5 lower to 0.51 higher) | ⊕⊕⊕⊕  HIGH | IMPORTANT |

^1^ The sample size was small

^2^ Funnel plot can not be judged, and there was no related interest statement

**Question:** Should RT+tDCS vs RT+FES+SS be used for lower Limb dysfunction after stroke?

| **Quality assessment** | | | | | | | **No of patients** | | **Effect** | | **Quality** | **Importance** |
| --- | --- | --- | --- | --- | --- | --- | --- | --- | --- | --- | --- | --- |
|  |  |  |  |  |  |  |  |  |  |  |  |  |
| **No of studies** | **Design** | **Risk of bias** | **Inconsistency** | **Indirectness** | **Imprecision** | **Other considerations** | **RT+tDCS** | **RT+FES+SS** | **Relative**  **(95% CI)** | **Absolute** |  |  |
| **10mMWS (Better indicated by higher values)** | | | | | | | | | | | | |
| 1 | randomised trials | serious^1^ | no serious inconsistency | no serious indirectness | serious^2^ | none | 11 | 12 | - | SMD 0.20 higher (0.62 lower to 1.02 higher) | ⊕⊕OO  LOW | IMPORTANT |

^1^ Subjects and study personnel were not blinded

^2^ The sample size was small
