# Supplementary material for: Optimization of electrical stimulation for the treatment of lower limb dysfunction after stroke: A systematic review and Bayesian network meta-analysis of randomized controlled trials
Source: PLoS One. 2023 May 11;18(5):e0285523. doi: 10.1371/journal.pone.0285523 (PMC10174537; doi:10.1371/journal.pone.0285523)
Supplement: S6 Table — (DOCX) [file pone.0285523.s006.docx]

**S7 Table.** Assessment of convergence of the model results.

1. PSRF value in FMA-LE.

| **Parameter** | **PSRF** |
| --- | --- |
| d.RT.RT+FES | 1 |
| d.RT.RT+NMES | 1 |
| d.RT.RT+SS | 1 |
| d.RT.RT+TEAS | 1 |
| d.RT.RT+TENS | 1 |
| d.RT+FES.RT+tDCS | 1 |
| d.RT+FES.RT+tDCS+FES | 1 |
| sd.d | 1 |

1. PSRF value in BBS.

| **Parameter** | **PSRF** |
| --- | --- |
| d.RT.RT+NMES | 1 |
| d.RT+FES.RT | 1 |
| d.RT+FES.RT+SS | 1 |
| d.RT+FES.RT+tDCS | 1.01 |
| d.RT+FES.RT+tDCS+FES | 1 |
| d.RT+SS.RT+TEAS | 1 |
| d.RT+tDCS+FES.RT+FES+SS | 1 |
| sd.d | 1 |

1. PSRF value in MBI.

| **Parameter** | **PSRF** |
| --- | --- |
| d.RT.RT+FES | 1 |
| d.RT.RT+NMES | 1 |
| d.RT.RT+TEAS | 1 |
| d.RT+FES.RT+tDCS | 1 |
| d.RT+FES.RT+tDCS+FES | 1 |
| sd.d | 1 |

1. PSRF value in 10mMWS(1).

| **Parameter** | **PSRF** |
| --- | --- |
| d.RT.RT+FES | 1 |
| d.RT.RT+TENS | 1 |
| sd.d | 1 |

1. PSRF value in 10mMWS(2).

| **Parameter** | **PSRF** |
| --- | --- |
| d.RT+FES+SS.RT+tDCS | 1 |
| d.RT+FES+SS.RT+tDCS+FES | 1 |
| sd.d | 1 |

1. PSRF value in CSS.

| **Parameter** | **PSRF** |
| --- | --- |
| d.RT.RT+FES | 1.01 |
| d.RT.RT+SS | 1 |
| d.RT.RT+TEAS | 1 |
| sd.d | 1 |
