# Supplementary material for: Optimization of electrical stimulation for the treatment of lower limb dysfunction after stroke: A systematic review and Bayesian network meta-analysis of randomized controlled trials
Source: PLoS One. 2023 May 11;18(5):e0285523. doi: 10.1371/journal.pone.0285523 (PMC10174537; doi:10.1371/journal.pone.0285523)
Supplement: S7 Table — (DOCX) [file pone.0285523.s007.docx]

**S9 Table.** Treatment ranking and SUCRA for outcome data.

(1)Treatment ranking and SUCRA for FMA-LE.

| **Treatment** | **SUCRA** | **PrBest** | **MeanRank** |
| --- | --- | --- | --- |
| RT+tDCS+FES | 76.4 | 37.7 | 2.6 |
| RT+FES | 64.9 | 3.5 | 3.5 |
| RT+tDCS | 84.8 | 44.8 | 2.1 |
| RT+TEAS | 56.7 | 6.0 | 4.0 |
| RT+TENS | 31.8 | 1.6 | 5.8 |
| RT+SS | 27.8 | 1.6 | 6.1 |
| RT+NMES | 48.9 | 4.7 | 4.6 |
| RT | 8.7 | 0.0 | 7.4 |

(2)Treatment ranking and SUCRA for MBI.

| **Treatment** | **SUCRA** | **PrBest** | **MeanRank** |
| --- | --- | --- | --- |
| RT+tDCS+FES | 73.2 | 36.6 | 2.3 |
| RT+FES | 51.7 | 2.4 | 3.4 |
| RT+tDCS | 81.1 | 42.8 | 1.9 |
| RT+TEAS | 23.9 | 2.3 | 4.8 |
| RT+NMES | 61.6 | 15.9 | 2.9 |
| RT | 8.5 | 0.0 | 5.6 |

(3)Treatment ranking and SUCRA for BBS.

| **Treatment** | **SUCRA** | **PrBest** | **MeanRank** |
| --- | --- | --- | --- |
| RT+tDCS+FES | 91.5 | 58.2 | 1.6 |
| RT+FES | 63.7 | 1.6 | 3.5 |
| RT+tDCS | 82.8 | 26.6 | 2.2 |
| RT+TEAS | 61.0 | 12.5 | 3.7 |
| RT+SS | 14.6 | 0.0 | 7.0 |
| RT+NMES | 45.5 | 1.0 | 4.8 |
| RT+FES+SS | 29.9 | 0.1 | 5.9 |
| RT | 11.0 | 0.0 | 7.2 |

(4)Treatment ranking and SUCRA for 10mMWS(1).

| **Treatment** | **SUCRA** | **PrBest** | **MeanRank** |
| --- | --- | --- | --- |
| RT+FES | 82.3 | 65.5 | 1.4 |
| RT+TENS | 52.8 | 34.1 | 1.9 |
| RT | 14.9 | 0.4 | 2.7 |

(5)Treatment ranking and SUCRA for 10mMWS(2).

| **Treatment** | **SUCRA** | **PrBest** | **MeanRank** |
| --- | --- | --- | --- |
| RT+tDCS+FES | 44.2 | 24.6 | 2.1 |
| RT+tDCS | 59.2 | 49.8 | 1.8 |
| RT+FES+SS | 46.6 | 25.6 | 2.1 |

(6)Treatment ranking and SUCRA for 10mMWS(2).

| **Treatment** | **SUCRA** | **PrBest** | **MeanRank** |
| --- | --- | --- | --- |
| RT+FES | 82.7 | 48.2 | 1.5 |
| RT+TEAS | 83.9 | 51.8 | 1.5 |
| RT+SS | 25.3 | 0.0 | 3.2 |
| RT | 8.1 | 0.0 | 3.8 |
