# Supplementary material for: Optimization of electrical stimulation for the treatment of lower limb dysfunction after stroke: A systematic review and Bayesian network meta-analysis of randomized controlled trials
Source: PLoS One. 2023 May 11;18(5):e0285523. doi: 10.1371/journal.pone.0285523 (PMC10174537; doi:10.1371/journal.pone.0285523)

**S6 Fig.** Pairwise Meta-Analysis.

FMA(RT+FES vs RT)


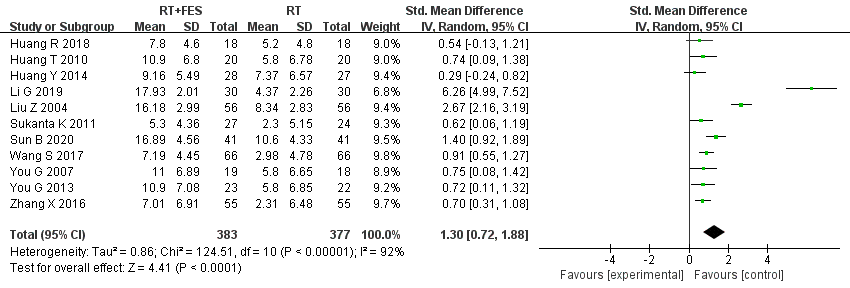


FMA(RT+FES vs RT+SS)


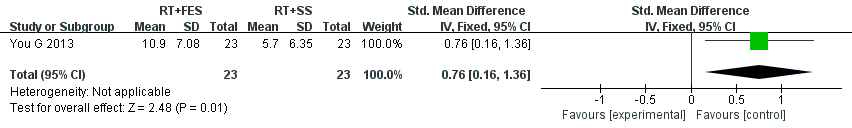


FMA(RT+FES vs RT+tDCS)


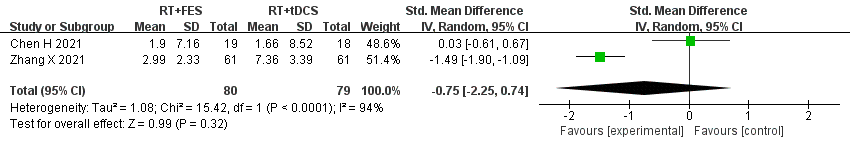


FMA(RT+NMES vs RT)


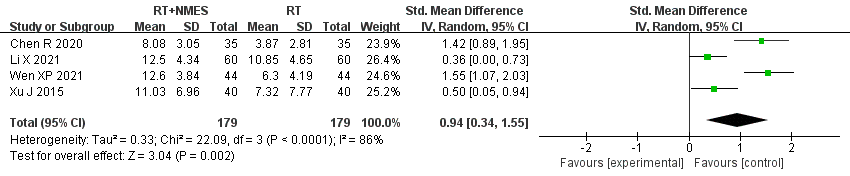


FMA(RT+SS vs RT)


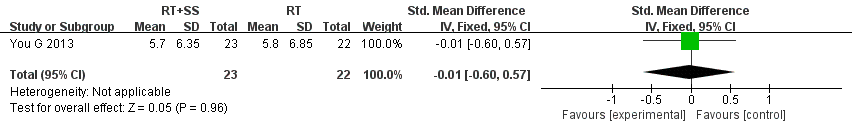


FMA(RT+tDCS+FES vs RT+FES)


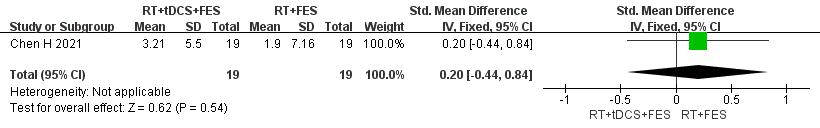


FMA(RT+tDCS+FES vs RT+tDCS)


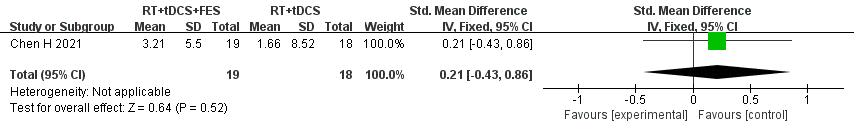


FMA(RT+TEAS vs RT)


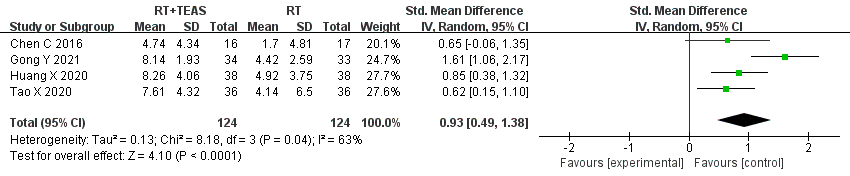


FMA(RT+TEAS vs RT+SS)


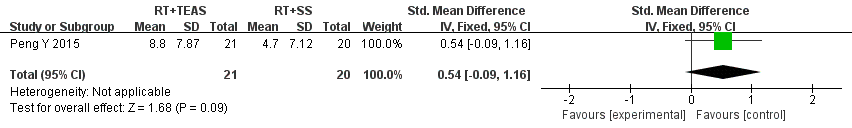


FMA(RT+TEAS vs RT+TENS)


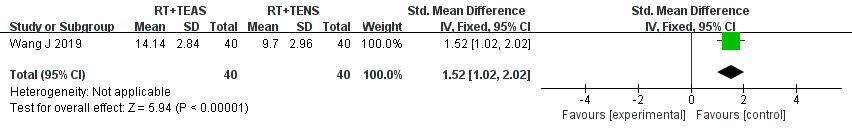


FMA(RT+TENS vs RT)


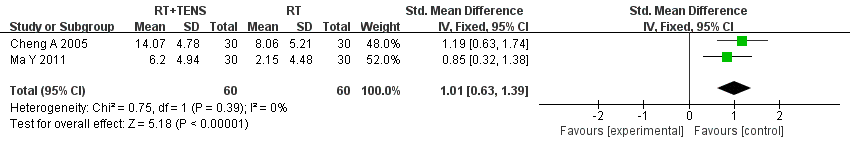


MBI(RT+FES vs RT)


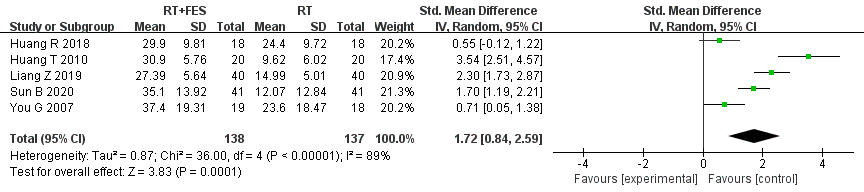


MBI(RT+FES vs RT+tDCS)


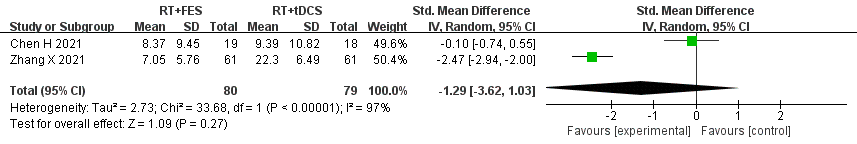


MBI(RT+NMES vs RT)


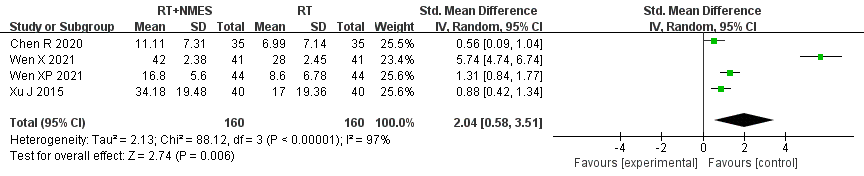


MBI(RT+tDCS+FES vs RT+FES)


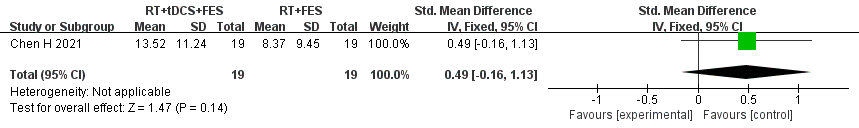


MBI(RT+tDCS+FES vs RT+tDCS)


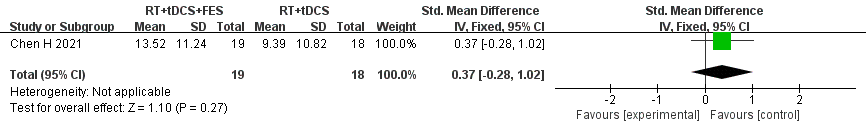


MBI(RT+TEAS vs RT)


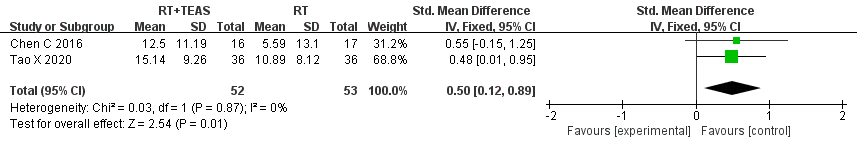


10mMWS(RT+FES vs RT)


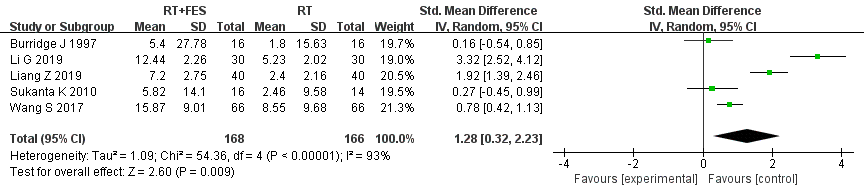


10mMWS(RT+tDCS vs RT+FES+SS)


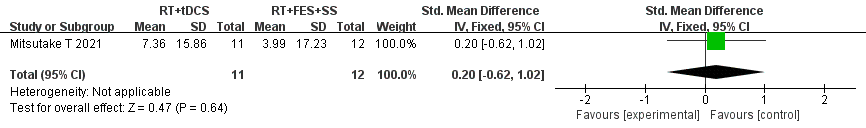


10mMWS(RT+tDCS+FES vs RT+FES+SS)


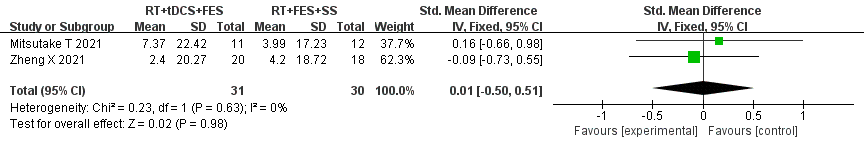


10mMWS(RT+tDCS+FES vs RT+tDCS)


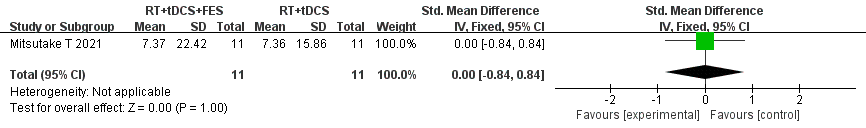


10mMWS(RT+TENS vs RT)


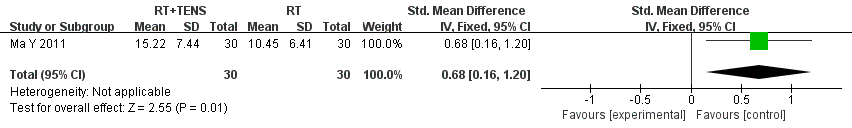


BBS(RT+FES vs RT)


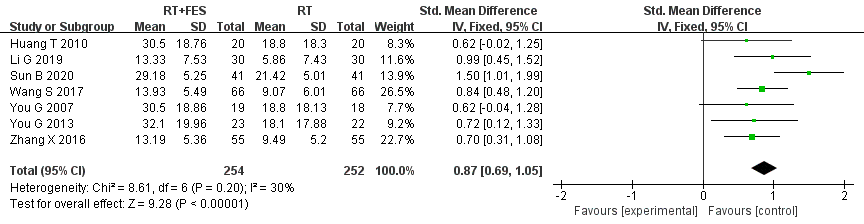


BBS(RT+FES vs RT+SS)


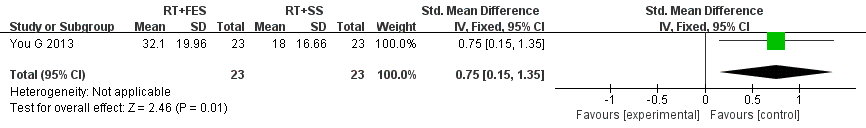


BBS(RT+FES vs RT+tDCS)


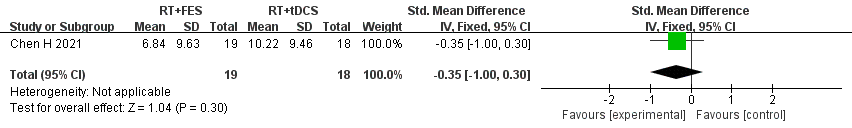


BBS(RT+NMES vs RT)


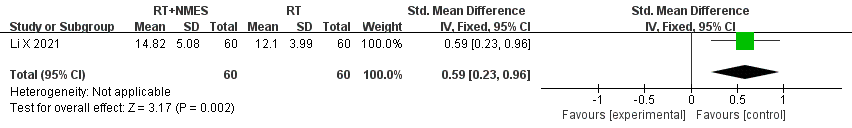


BBS(RT+SS vs RT)


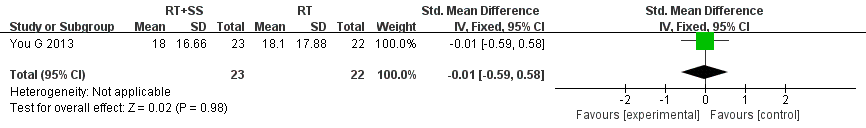


BBS(RT+tDCS+FES vs RT+FES)


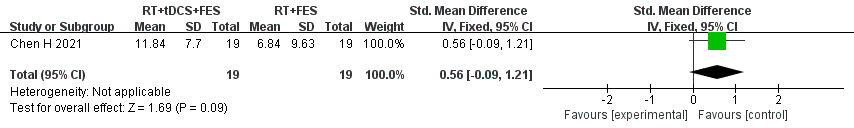


BBS(RT+tDCS+FES vs RT+FES+SS)


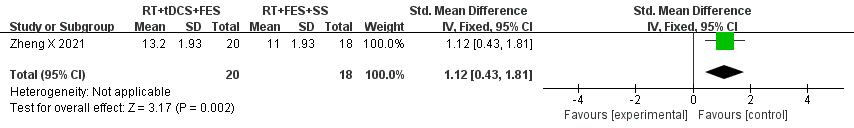


BBS(RT+tDCS+FES vs RT+tDCS)


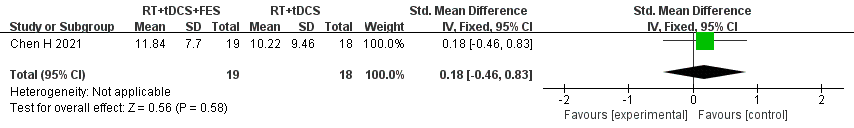


BBS(RT+TEAS vs RT+SS)


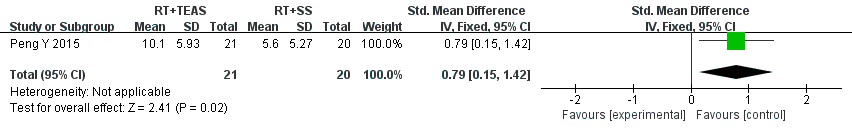


CSS(RT+FES vs RT)


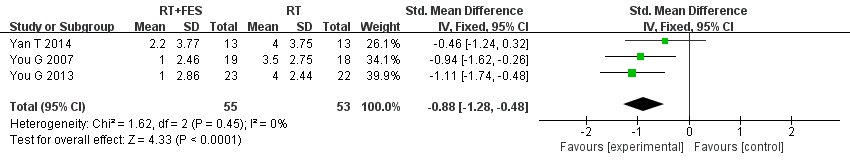


CSS(RT+FES vs RT+SS)


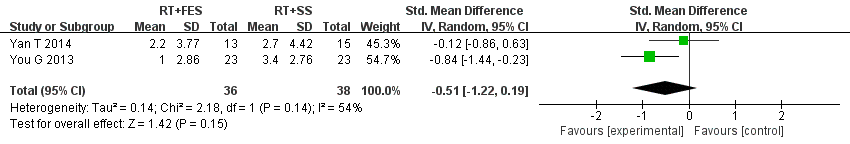


CSS(RT+SS vs RT)


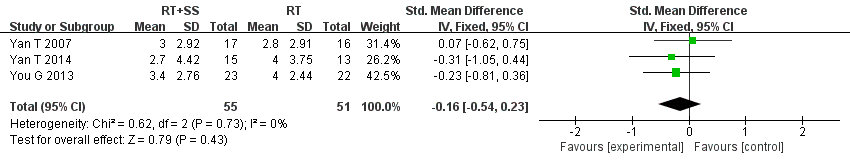


CSS(RT+TEAS vs RT)


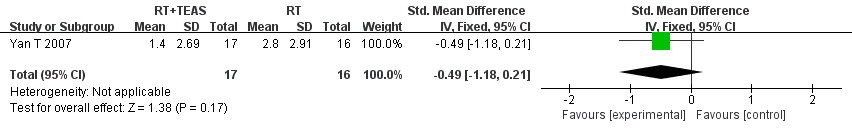


CSS(RT+TEAS vs RT+SS)


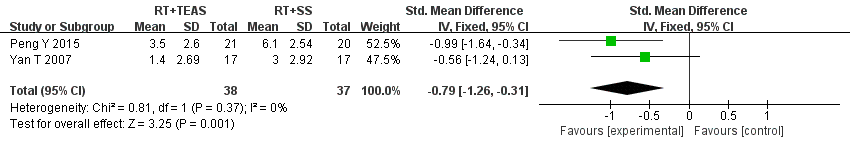

Supplement: S1 Fig — (DOCX) [file pone.0285523.s008.docx]
