# Supplementary material for: Optimization of electrical stimulation for the treatment of lower limb dysfunction after stroke: A systematic review and Bayesian network meta-analysis of randomized controlled trials
Source: PLoS One. 2023 May 11;18(5):e0285523. doi: 10.1371/journal.pone.0285523 (PMC10174537; doi:10.1371/journal.pone.0285523)

**S8 Fig.** Ranking probability.

**Ranking probability figure for reduction in FMA-LE**

**
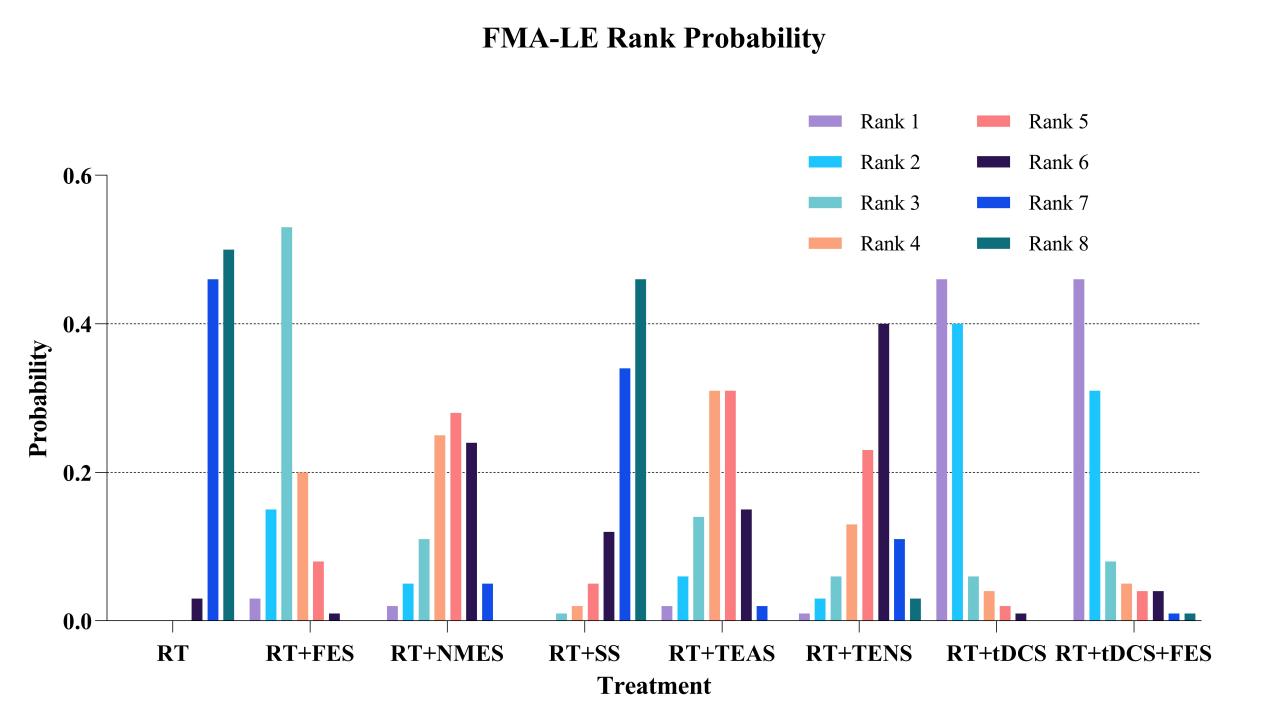
**

**Ranking probability figure for reduction in BBS**

**
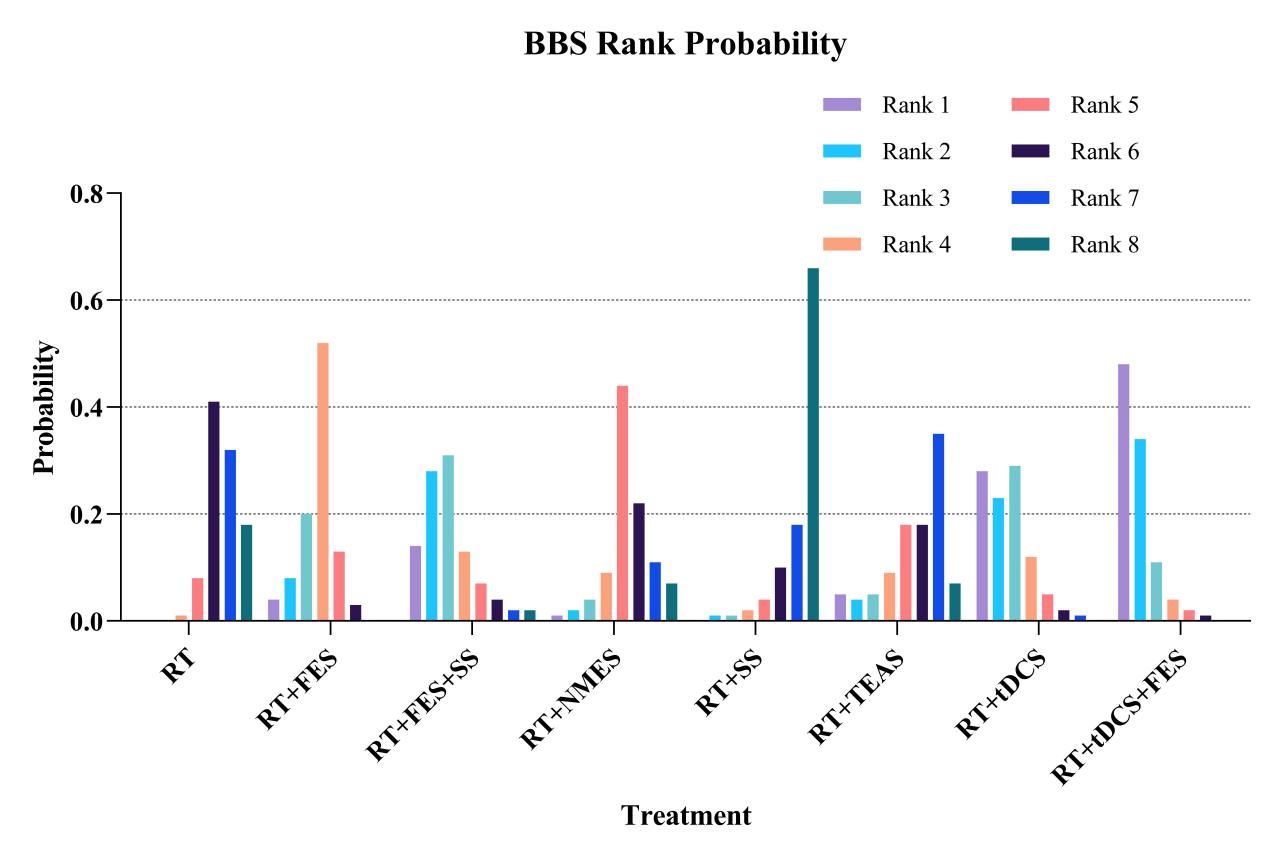
**

**Ranking probability figure for reduction in MBI**

**
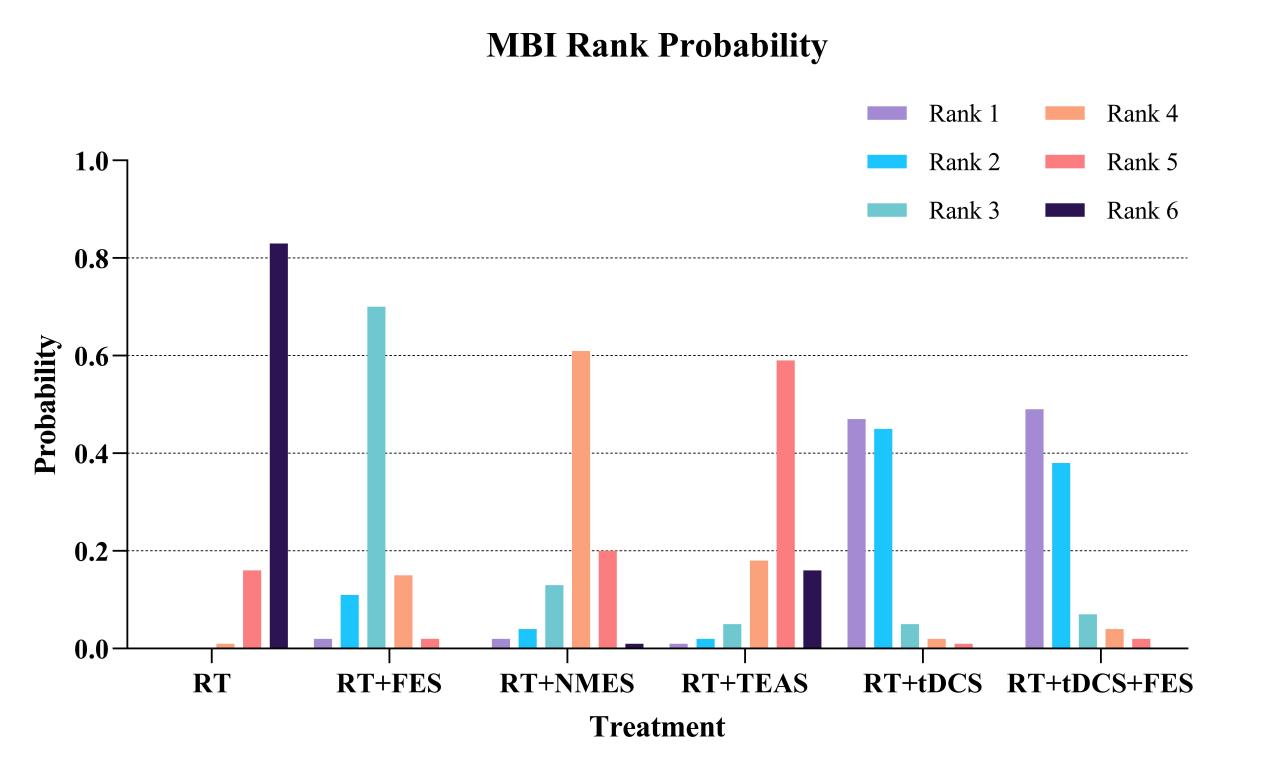
**

**Ranking probability figure for reduction in CSS**

**
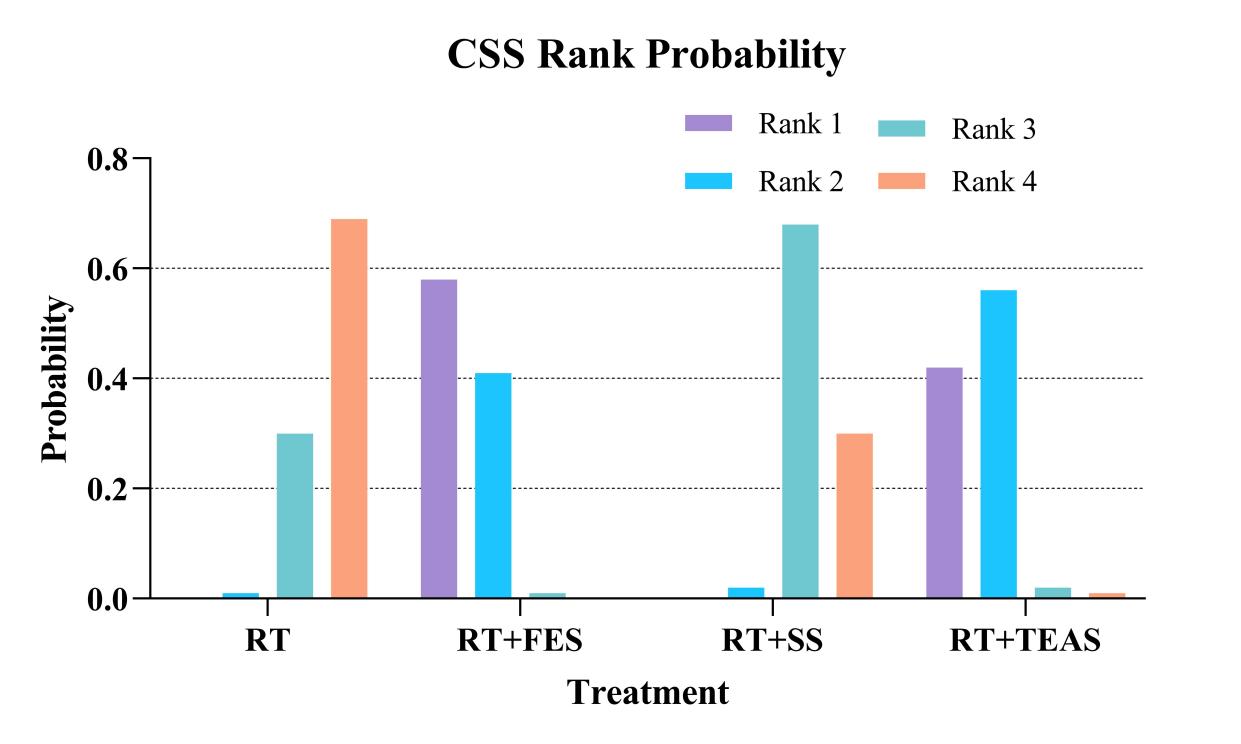
**

**Ranking probability figure for reduction in 10mMWS(1)**

**
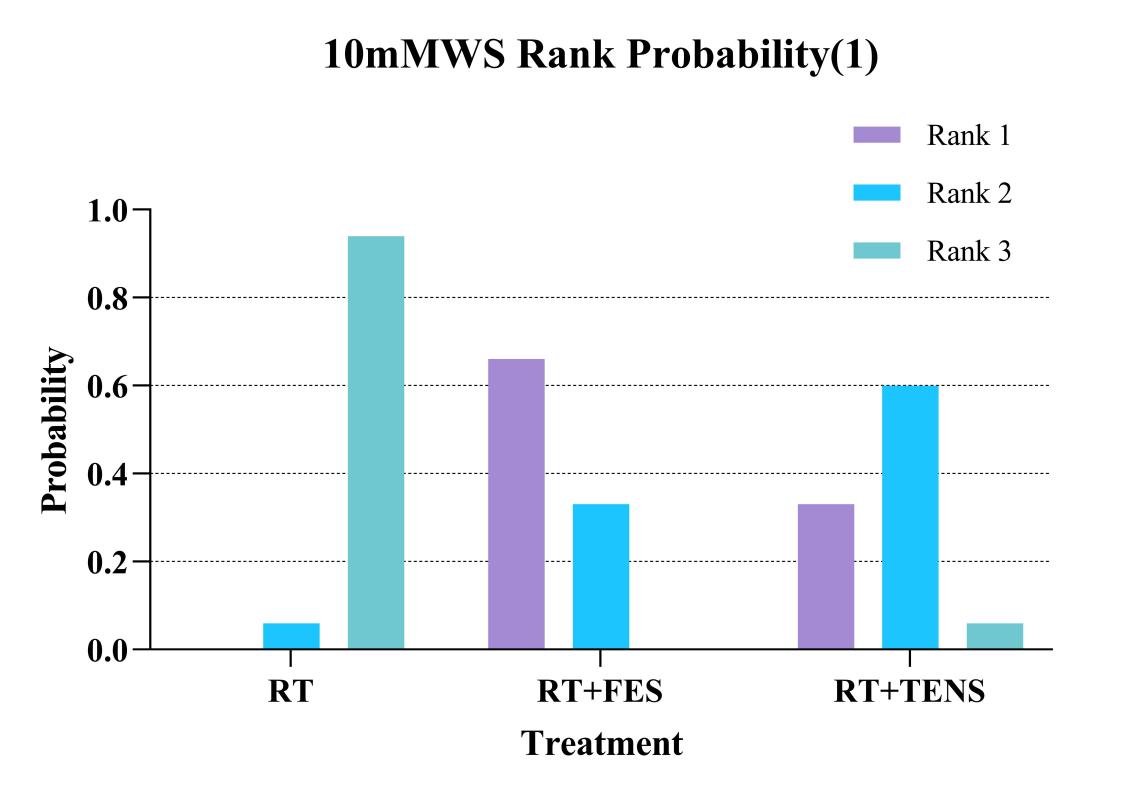
**

**Ranking probability figure for reduction in 10mMWS(2)**


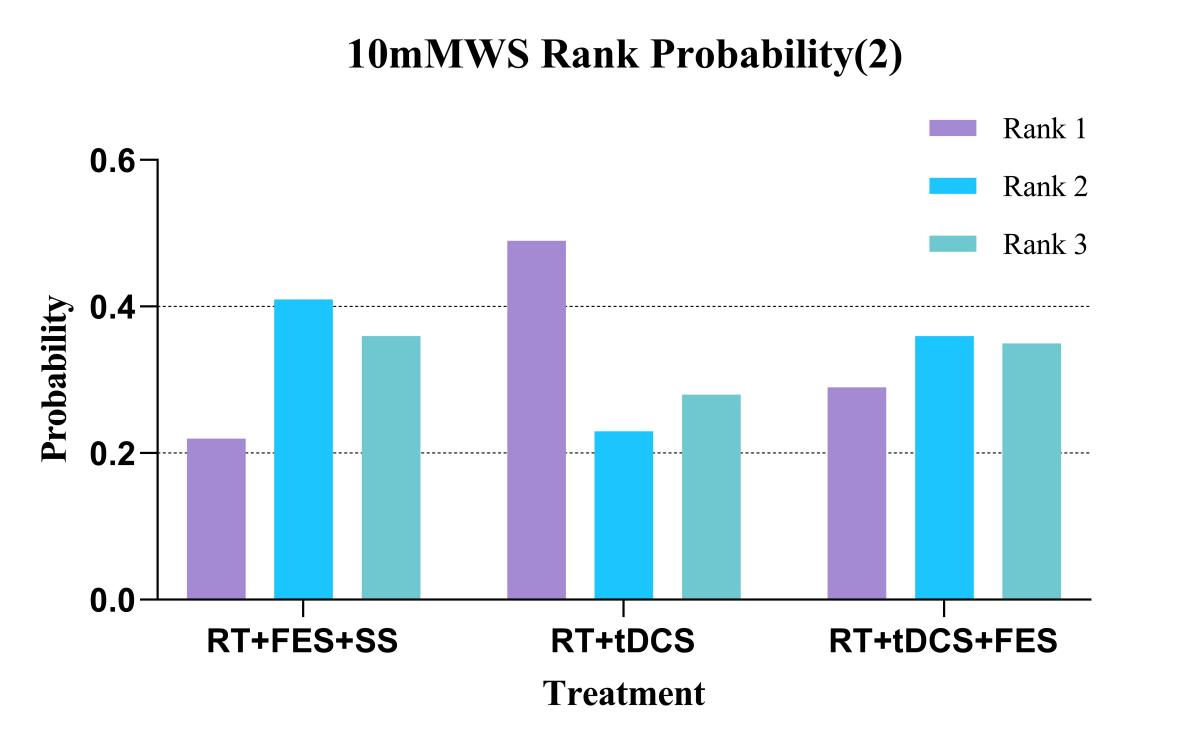

Supplement: S2 Fig — (DOCX) [file pone.0285523.s009.docx]
